# Distribution, characterization, and evolution of heavy metal resistance genes and Tn7-like associated heavy metal resistance Gene Island of *Burkholderia*

**DOI:** 10.3389/fmicb.2023.1252127

**Published:** 2023-11-23

**Authors:** Yanhong Lan, Meijia Liu, Yao Song, Yu Cao, Fosheng Li, Dening Luo, Dairong Qiao

**Affiliations:** ^1^Microbiology and Metabolic Engineering Key Laboratory of Sichuan Province, Key Laboratory of Bio-Resources and Eco-Environment of Ministry of Education, College of Life Sciences, Sichuan University, Chengdu, Sichuan, China; ^2^Chengdu University of Information Technology, Chengdu, China

**Keywords:** *Burkholderia*, comparative genome, pan-genome, heavy metal resistance gene, Tn7-like transposon, horizontal transfer

## Abstract

**Introduction:**

*Burkholderia* is a rod-shaped aerobic Gram-negative bacteria with considerable genetic and metabolic diversity, which can beused for bioremediation and production applications, and has great biotechnology potential. However, there are few studies on the heavy metal resistance of the *Burkholderia* genus.

**Methods:**

In this paper, the distribution, characteristics and evolution of heavy metal resistance genes in *Burkholderia* and the gene island of Tn7-like transposable element associated with heavy metal resistance genes in *Burkholderia* were studied by comparative genomic method based on the characteristics of heavy metal resistance.

**Results and discussion:**

The classification status of some species of the Burkholderia genus was improved, and it was found that Burkholderia dabaoshanensis and Burkholderia novacaledonica do not belong to the Burkholderia genus.Secondly, comparative genomics studies and pan-genome analysis found that the core genome of Burkholderia has alarger proportion of heavy metal resistance genes and a greater variety of heavy metalresistance genes than the subsidiary genome and strain specific genes. Heavy metal resistance genes are mostly distributed in the genome in the form of various gene clusters (for example, mer clusters, ars clusters, czc/cusABC clusters). At the same time, transposase, recombinase, integrase and other genes were foundupstream and downstream of heavy metal gene clusters, indicating that heavy metal resistance genes may beobtained through horizontal transfer. The analysis of natural selection pressure of heavy metal resistance genes showed that heavy metal resistance genes experienced strong purification selection under purification selection pressure in the genome.The Tn7 like transposable element of Burkholderia was associated with the heavy metal resistance gene island, and there were a large number of Tn7 transposable element insertion events in genomes. At the same time, BGI metal gene islands related to heavy metal resistance genes of Tn7 like transposable element were found, and these gene islands were only distributed in Burkholderia cepacia, Burkholderia polyvora, and Burkholderia contaminant.

## Introduction

With the development of industrialization and the interference of natural biogeochemical cycle, heavy metal (such as cadmium, lead, arsenic, mercury) pollution poses a serious threat to natural ecosystems and human health (Ali et al., 2013; Vignaroli et al., 2018). Unlike organic pollutants, heavy metals cannot be degraded and difficult to remove, leading to persistent environmental hazards, which can lead to microbial and plant poisoning in the environment, affecting growth and development or death, and even affecting the normal operation of ecosystems in some areas, thereby exacerbating the harm to the environment.

Heavy metal tolerant bacteria can survive in the environment polluted by heavy metals, and can be isolated and selected for bioremediation of contaminated sites. Multiple microorganisms have been reported to have resistance to heavy metals. For example, the impact of plant growth promoting (PGP) bacteria on plant growth had received much attention in phytoremediation (Das et al., 2014; Hao et al., 2015; Chen et al., 2016). PGP bacteria can ingest the genus *Phyllostachys* and promote plant growth through various mechanisms (Yuan et al., 2018). Many PGP bacteria that are resistant to specific heavy metals such as cadmium, nickel, and arsenic promote metal absorption and transport, or improve plant growth and heavy metal tolerance (Das et al., 2014; Wang et al., 2022).

*Burkholderia* is a ubiquitous microorganism with high resistance to heavy metals. In recent years, many new species of *Burkholderia* have been tested and analyzed, and it is found that some *Burkholderia* have high efficiency in biological pest control and bioremediation (Coenye and Vandamme, 2003; Pal et al., 2022). *Burkholderia* sp. J62 polluted soil absorbs lead and cadmium, while also promoting plant growth (Jiang et al., 2008). The *Burkholderia* sp. Z-90 fermentation broth was used for bioleaching remediation of heavy metal contaminated soil. The removal rate of zinc was 44.0%, lead 32.5%, manganese 52.2%, cadmium 37.7%, copper 24.1%, and arsenic 31.6% (Yang et al., 2016). *Burkholderia* sp. LD-11 can accumulate copper and lead in contaminated soil, and produce indole-3-acetic acid, 1-aminonenenebb cyclopropane 1-carboxylate deaminase and iron carrier, which can increase the dry weight of plants growing in copper or lead contaminated soil, and increase the diversity of active soil urease and rhizosphere bacteria (Huang et al., 2013). *Burkholderia dabaoshanensis* GIMN1.004 was a new strain isolated from Dabaoshan, China, with strong cadmium tolerance (22 mmol/L) and adsorption capacity (144.94 mg/g) (Zhu et al., 2012). Ion gallium (III) had antibacterial activity against both Gram-negative and Gram positive bacteria as well as mycobacteria (Peeters et al., 2008; Choi et al., 2014, 2019; Tyrrell et al., 2015). Some studies had shown that resistance genes in microbial bacteria can repair pollution points in urban rivers, thereby achieving a sustainable green strategy for the environment (Lechuga-Ballesteros et al., 2009; Fu et al., 2023; Li et al., 2023; Tripathi et al., 2023).

The IrlR-IrlS two-component system of *B. pseudoallei* AJ1D8 was involved in the regulation of heavy metal resistance (Jones et al., 1997). IrlR and IrlS were homologous to two-component sensor responder proteins involved in regulating resistance to heavymetals. IrlR and IrlS may be involved in theregulation of two distinct phenotypes, invasion and heavymetal (Cd21 and Zn21) resistance. The dsbA-dsbB system of *B. cepacia* was involved in the production of protease and alkaline phosphatase, mobility, formation of metal efflux system (resistant to divalent cadmium and zinc ions) and multiple drug resistance (β-lactam, kanamycin, erythromycin, neomycin, ofloxacin and sodium dodecyl sulfate) (Hayashi et al., 2000). DsbA, the disulfide bond catalyst of *Escherichia coli*, is a periplasmic protein having a thioredoxin-like Cys-30-Xaa-Xaa-Cys-33 motif. DsbB, an integral membrane protein having two pairs of essential cysteines, reoxidizes DsbA that has been reduced upon functioning.

The *Burkholderia* genus had received widespread attention due to its pathogenicity and drug resistance, resulting in the accumulation of a large number of gene/proteome and various biochemical and physiological research results. At present, research on this genus is still focused on pathogenicity and drug resistance, while its environmental value, such as the adsorption of heavy metals (zinc, lead, manganese, chromium, etc.) in soil, is rarely studied. Moreover, the studies of this genus focused on the pathogenic gene island, and the heavy metal resistance still was not reported. Therefore, understanding the resistance of the *Burkholderia* genus to heavy metals and the structure and characteristics of heavy metal islands in the genome is crucial for subsequent theoretical research and environmental adaptation of the genus.

## Materials and methods

### Source of genome data, genome filtering, and evaluation

220 plasmid sequences and 1841 complete or draft genomes of the *Burkholderia* genus were obtained from the national center for biotechnology information database (NCBI),^1^ involving a total of 32 bacterial species (Supplementary Table S1). 162,516 protein sequences were identified by CD hit (with a cutoff value of 0.8 and a sequence identity value of 0.9) (Li and Godzik, 2006). The ORFs, protein coding genes, and non-coding RNAs of the genome were predicted using prokka (Seemann, 2014). To select representative genomes and ensure accurate subsequent analysis, genomes or proteomic integrity below 85% integrity were filtered and evaluated using BUSCO (Simao et al., 2015), The average nucleotide similarity among strains with genome numbers greater than 3 was calculated by pyani (v0.2.7) software.

### Phylogenetic analysis

To understand the distribution of heavy metal resistance genes in *Burkholderia*, multiple sequence alignments of all selected 16S rDNA sequences were performed using the ClustalW method with default parameters. The neighbor-joining (NJ) method was used to construct phylogenetic trees of 16S rDNA sequences using the MEGA software (v7.0.26, with the following parameters: Poisson correction, pairwise deletion and a bootstrap test of 1,000 replicates) (Kumar et al., 2018). Some genomes do not contained 16S rDNA sequences, the Neighbor Joining method of CVTree3 (Zuo and Hao, 2015) was used to construct a phylogenetic tree based on the entire genome (Set the K value to 5, 6, and 7). After analyzing pan-genome, the MLST method (Multi Locus Sequence Typing) of BPGA was used to obtain a phylogenetic tree based on the core genome, and the final results were visualized using iTOL online tools (Letunic and Bork, 2016).

### Genomic identification of toxic heavy metal resistance

The filtered 1831 genomes were compared using BLASTP to the BacMet database, and genes related to heavy metal resistance in the genome of *Burkholderia* were identified. The BacMet dataset used for analysis was the experimentally confirmed gene set for metal resistance function (BacMet Experimental Database) (e < 1e-6, sequence similarity ≥40%, sequence coverage ≥55%, alignment amino acid length ≥ 80). Multiple sequence alignments of the nucleic acid sequence of heavy metal resistance genes were performed using the ClustalW method with default parameters. The phylogenetic trees were constructed using the MEGA software (Kumar et al., 2018).

### Pan-genome analysis and speculation of *Burkholderia* model

The bacterial pan-genome analysis (BPGA) process was used to identify the orthologous genome of the genome of *Burkholderia* experimental data and to infer the pan-genome model of *Burkholderia*. In this study, the pan-genome and core genome size of *Burkholderia* were inferred by the intrinsic function of BPGA. The calculation formula of BPGA’s intrinsic function is as follows:


$$N_{\mathrm{pan}}=\sum_{i=1}^{n}f_{\mathrm{pan}}\left(Gi\right)$$

$$N_{\mathrm{core}}=\sum_{i=1}^{n}f_{\mathrm{core}}\left(Gi\right)$$

Gi represents the ith gene family, n was the total number of different gene family obtained from the entire dataset and the pan/core genome size, and (Npan/Core) represents the size of the pan/core genome after the n th genome was added from the dataset.

The power law regression model of pan-genome data and the exponential curve fitting model of core genome data were as follows:


$$Y_{\mathrm{pan}}=A_{\mathrm{pan}}.x^{B_{\mathrm{pan}}}+C_{\mathrm{pan}}$$

$$Y_{\mathrm{core}}=A_{\mathrm{core}}.x^{B_{\mathrm{core}}}+C_{\mathrm{core}}$$

A_pan_, B_pan_, C_pan_, and A_core_, B_core_, C_core_ were fitting parameters. Y_pan_ and Y_core_ were used to calculate the size of pan-genome and core genome size, respectively. If B_pan_ < 0, it means that pan-genome was closed. With the increase of additional genomes, the size of pan-genome reached a constant value.

### Prediction of selection pressure

The ratio of nucleotide non synonymous substitution (dN) and synonymous substitution (dS) of each heavy metal lineal homeotic gene was estimated to evaluate the strength of natural selection of heavy metal resistance genes in the evolution of *Burkholderia*. Muscle (v3.8.1551) (Edgar, 2004) was used for various heavy metal resistant protein alignments, converting the aligned protein sequences into corresponding nucleotide alignments using pal2nal (v14) (Suyama et al., 2006), with an output format of paml. Finally, the codeml program of the paml (v4.9) (Yang, 2007) software toolkit was use to calculate the dN and dS values, and select the branch model (with the main parameters set to CodonFreq = 2, model = 2, NSsites = 0) to calculate the Ka, Ks, and ω value. The non synonymous substitution rate (dN)/ synonymous substitution rate (dS) ratios were counted using DnaSP.^2^ It was generally believed that positive selection results in dN/dS > 1, while negative (purified) selection results in dN/dS < 1.

### Identification and insertion location of Tn7 like transposable element

The structural domains of various proteomes in the *Burkholderia* genus were annotated by interprescan (Jones et al., 2014). Candidate proteins were obtained (TnsA (PF08722, PF08721), TnsB (PF00665, PF09299), TnsC (PF13401), TnsD (PF15978), TniQ (PF06527)). The nucleic acid sequences containing TnsA, TnsB, TnsC, and TnsD (TniQ) were directly identified as 5 – bp target site repeats based on the left and right terminal characteristics of Tn7, the 8 – bp terminal sequence ended of 5 ‘- TGT-3’/3 ‘- ACA-5’, and a 22 bp TnsB binding sequence (Gary et al., 1996; Choi et al., 2014; Kaczmarska et al., 2022). There were three TnsB binding sequences spaced at the left end, with a length of ~150 bp, and four overlapping TnsB binding sequences at the right end, with a length of ~90 bp. To determine the location of Tn7 like transposable element structure inserted into the genome and the source of the inserted sequence, the complete Tn7-like and its upstream and downstream 5 kb sequences were online compared to NCBI blastn to find the location of the structure on other genomes or plasmids. Localize blastn alignment after localization to generate alignment file (output format outfmt 6), the GenBank file of Tn7-like transposable element was visualized using BacAnt (Hua et al., 2021).

### Identification of gene islands and functional annotation analysis of heavy metal resistance gene islands associated with Tn7 like transposable element

The gene islands of the genome were predicted by IslandViewer (Bertelli et al., 2017). The analysis of codon preference using R3.8 package took the following steps: using a self-made Perl script to slide window scan (5 Kb size, 2.5 Kb step size), and calculate codon usage frequencies of genes in each window. Subsequently, principal component analysis was performed on this matrix in R and plotted using the scores of the first two components. The KEGG pathway analysis of Tn7-like transposable element associated heavy metal resistance of gene-island was conducted with BlastKOALA, and the protein was KO annotated. GO functional enrichment analysis was visualized using WEGO 2.0 (Ye et al., 2018).

## Results

### Distribution and characteristics of heavy metal resistance genes in *Burkholderia* genus

**Figure 1:**
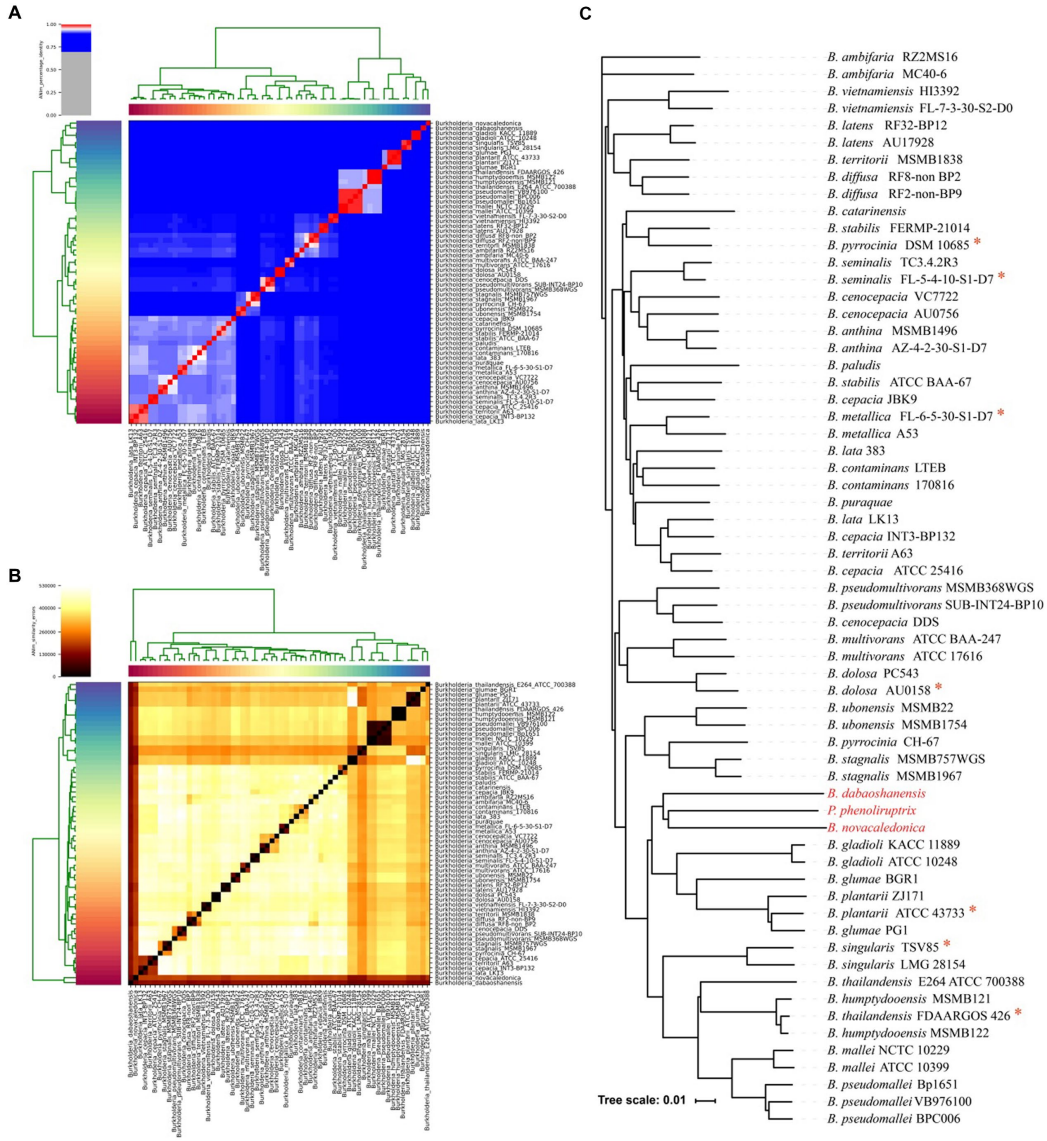
**(A)** Average nucleotide identity heat map of 62 *Burkholderia* genomes. **(B)** Average nucleotide error heat map of 62 *Burkholderia* genomes. **(C)** Genome-wide phylogenetic tree. The red * represents the model species.

A total of 220 plasmid sequences and 1841 complete or draft genomes of the *Burkholderia* genus were obtained from the NCBI database, containing a total of 32 bacterial species (Supplementary Table S2). After evaluating the integrity of the genome and proteome by BUSCO, 1831 genomes were obtained by filtering out incomplete genomes (integrity <85%). The genomic characteristics (GC-content, Genome size, genome integrity and protein integrity) of 1831 genomes were analyzed (Supplementary Figure S1). After average nucleotide similarity identification, 62 genomes of 32 species were used for pan-genome analysis (Supplementary Table S3). Previous reports have indicated that the percentage threshold for species boundaries is 95% ANI, and the similarity in ANI between 62 strains ranges from 84 to 99%. The average nucleotide similarity of *B. dabaoshanensis* and *B. novacaledonica* was lowest compared to other strains (approximately 84%, Figures 1A,B). The average nucleotide similarity between *Paraburkholderia* and *Burkholderia* is about 85%. Meanwhile, using the whole genome to construct a phylogenetic tree (Figure 1C), the results showed that *B. dabaoshanensis*, *B. novacaledonica*, and *P. phenoliruptrix* converged into one branch, and the 16 s rDNA phylogenetic tree results showed that *B. dabaoshanensis* and *B. novacaledonica* is individually aggregated into one branch (Supplementary Figure S2). Those results indicated that *B. dabaoshanensis* and *B. novacaledonica* do not belong to the *Burkholderia* genus, and these two strains were excluded in subsequent analysis.

### Evolutionary tree analysis and pan-genome analysis of *Burkholderia* genus

To analyze the genomic characteristics of each strain, the homologous groups of 62 strains were identified. It was found that the number of unique genes in *B. dabaoshanensis* and *B. novacaledonica* is greater than 1,000, with 1,609 and 1875, respectively. Combining the calculation results of average nucleotide similarity, the construction results of the whole genome phylogenetic tree, and the 16 s rDNA phylogenetic tree, it is once again confirmed that *B. dabaoshanensis* and *B. novacaledonica* does not belong to the *Burkholderia* genus.

**Figure 2:**
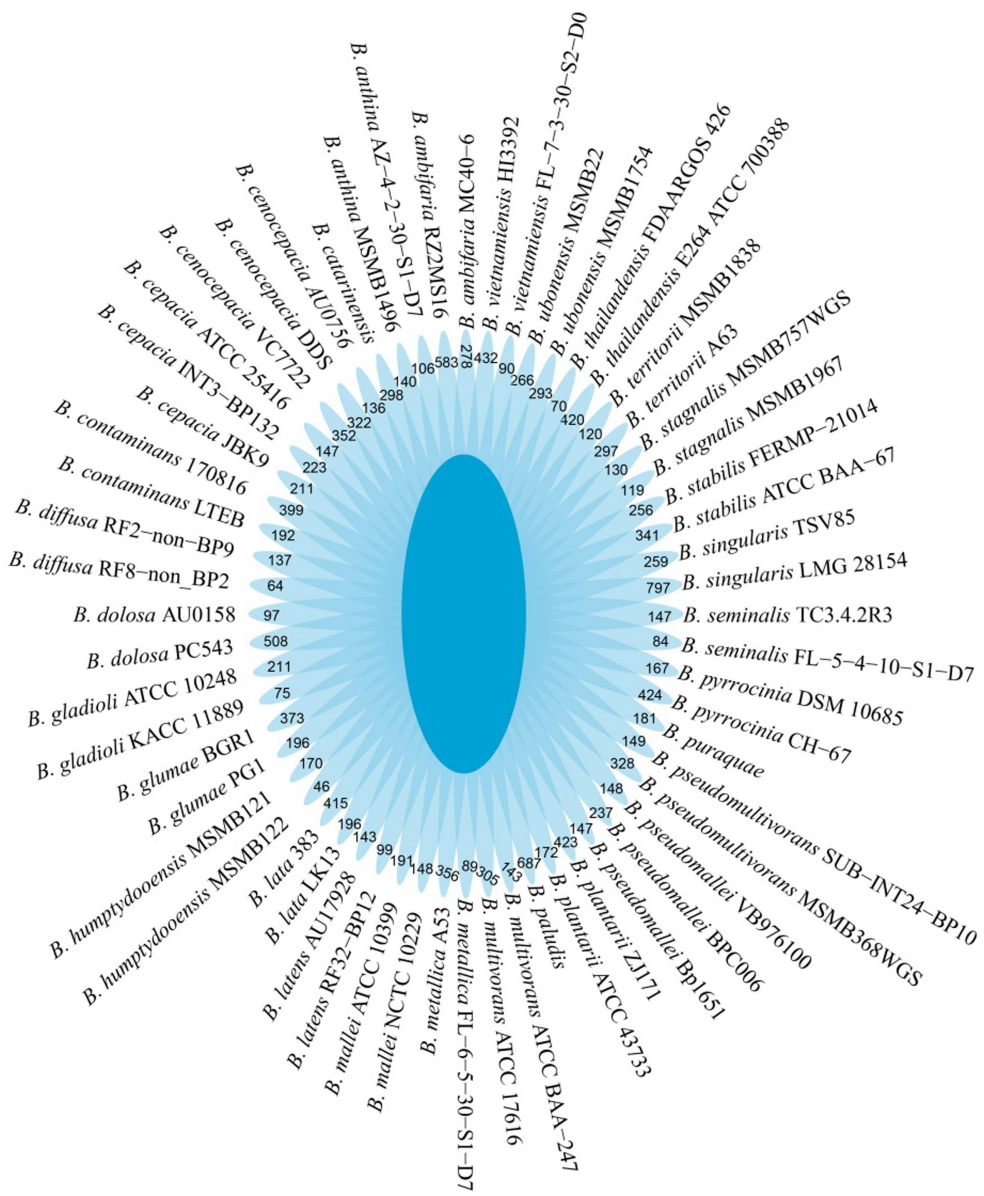
Petal diagram of the pangenome. The center is the number of orthologous coding sequences shared by all strains (i.e., the core genome). Numbers in nonoverlapping portions of each oval show the numbers of CDSs unique to each strain.

**Figure 3:**
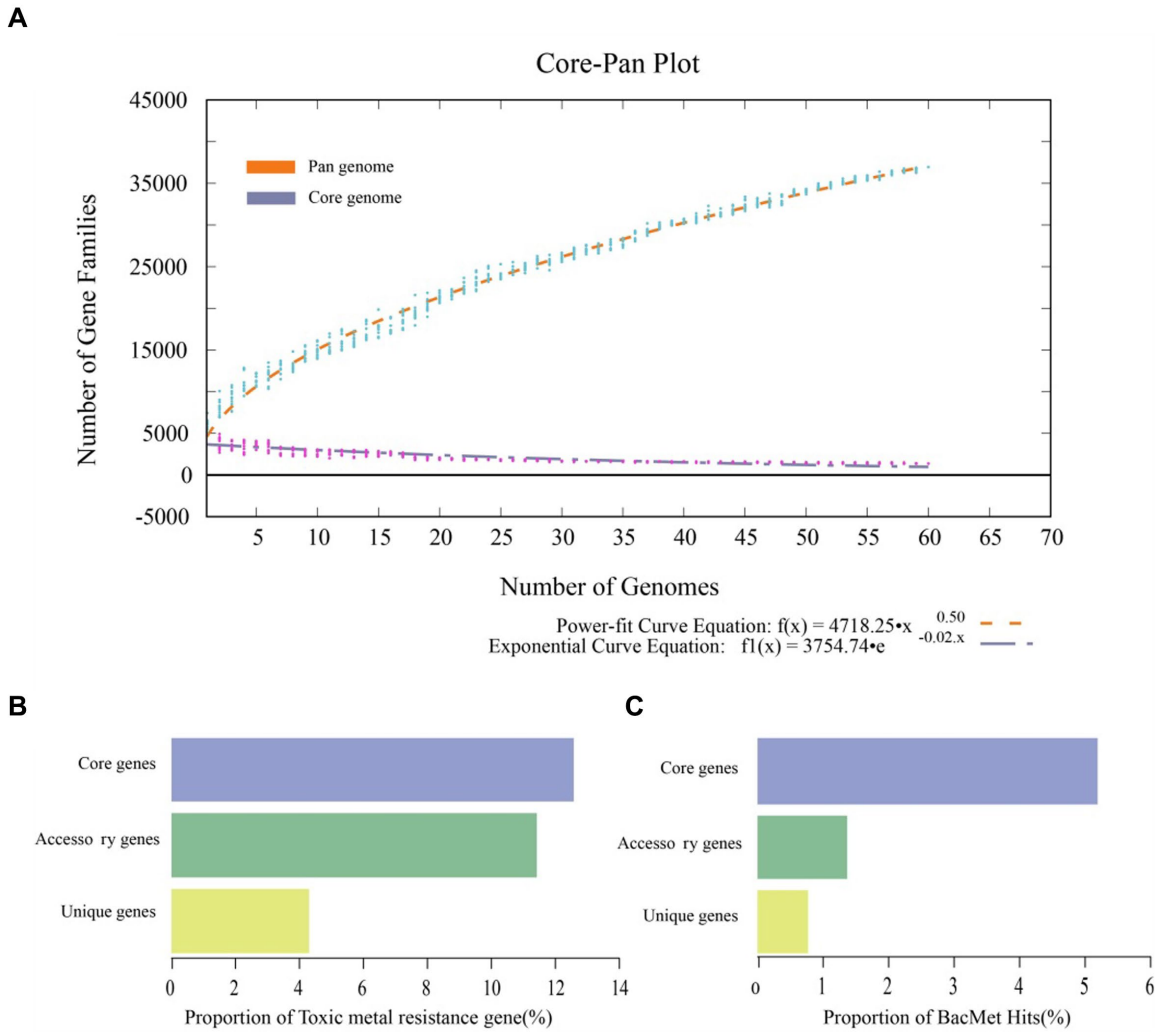
Pangenome analysis of *Burkholderia*. **(A)** Mathematical modeling of the pangenome and core genome. **(B)** Proportions of heavy metal resistance genes in pangenome, accessory genome, and unique genes. **(C)** Proportions of resistance genes to the BacMet database in the pangenome, accessory genome, and unique genes.

*B. dabaoshanensis* and *B. novacaledonica* were removed, and Pan-genome analysis was performed again for the remaining 60 genomes. The results showed that the number of protein coding genes of *Burkholderia* was 367,029. The unique genes of each strain range from 46 to 797 (Figure 2). The BacMet annotation results showed that 12.6, 11.4, and 4.3% of the genes in the core genome, accessory genome, and endemic genome were involved in heavy metal resistance and transformation, respectively (Figure 3A). The heavy metal annotation results of the core genome, accessory genome, and endemic genome showed that the heavy metal resistance genes in the core genome, accessory genome, and endemic genome account for 5.2, 1.3, and 0.8% of the BacMet database, respectively (Figures 3B,C). The surface core genome had more genes involved in heavy metal resistance than the affiliated and endemic genomes, and had more types of heavy metal resistance genes.

**Figure 4:**
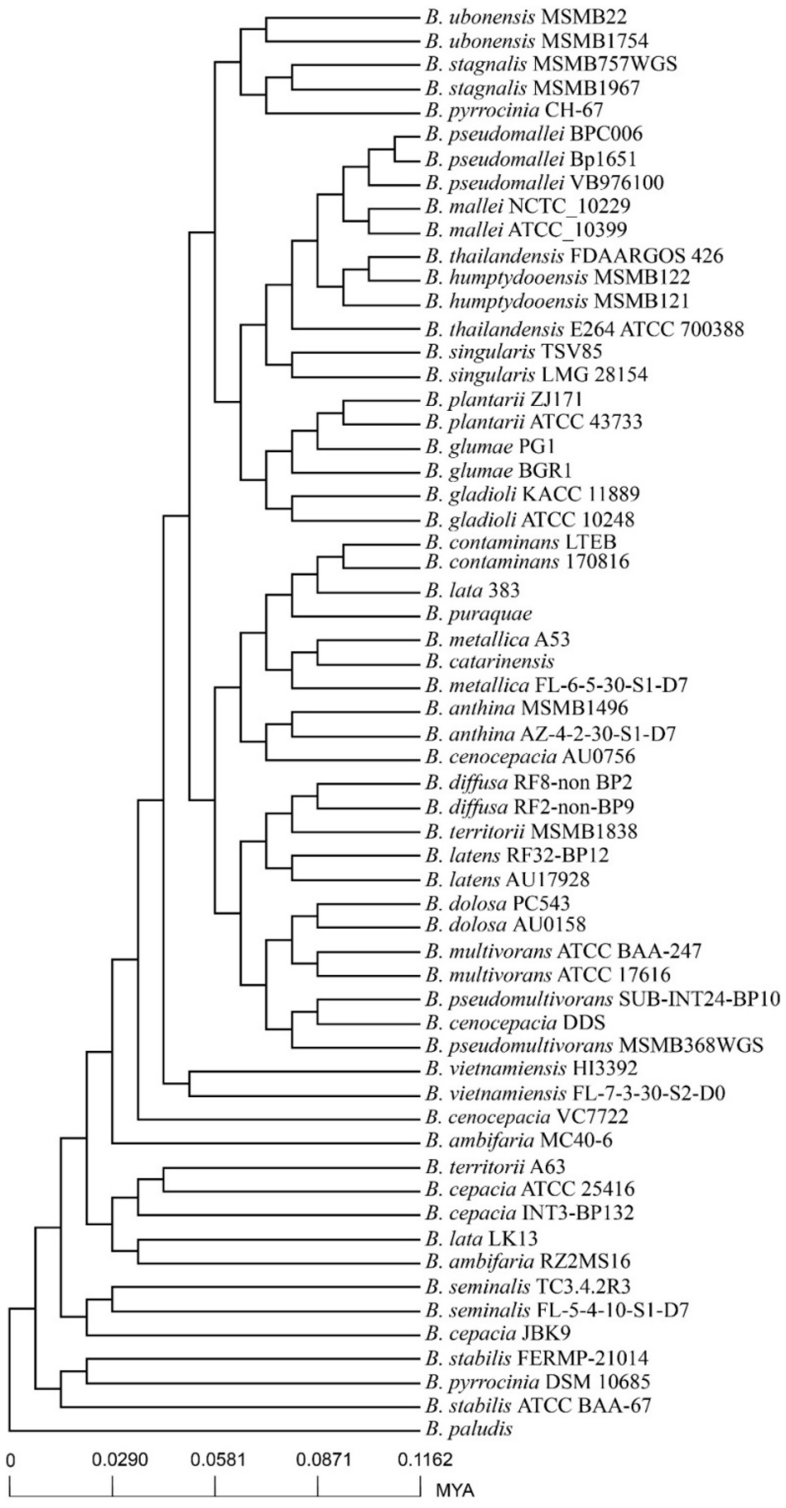
Divergence time trees of 60 *Burkholderia* genomes.

A phylogenetic tree was constructed using the core genome of 60 genomes. Compared to the previous whole genome phylogenetic tree results, the phylogenetic tree constructed based on core genes of 60 genomes was highly reliable (Figure 4). According to the core genome evolution results, *Burkholderia* first appeared 0.1162 million years ago (MYA, Million Years Ago) and differentiated into several species at nearly 0 ~ 0.05 MYA, indicating that the genus has been actively evolving in recent years. The power regression equation deduced by BPGA shows that B_pan_ = 0.503865, which belongs to the range of 0 < B_pan_ < 1, indicating that pan-genome is open. As the number of genomes increases, the extrapolation curve of the core genome followed a steep slope. As the number of genomes gradually was 60, the number of core genes tends to be relatively constant (Figure 3A).

### Distribution and evolution of heavy metal resistance genes in *Burkholderia* genus

**Figure 5:**
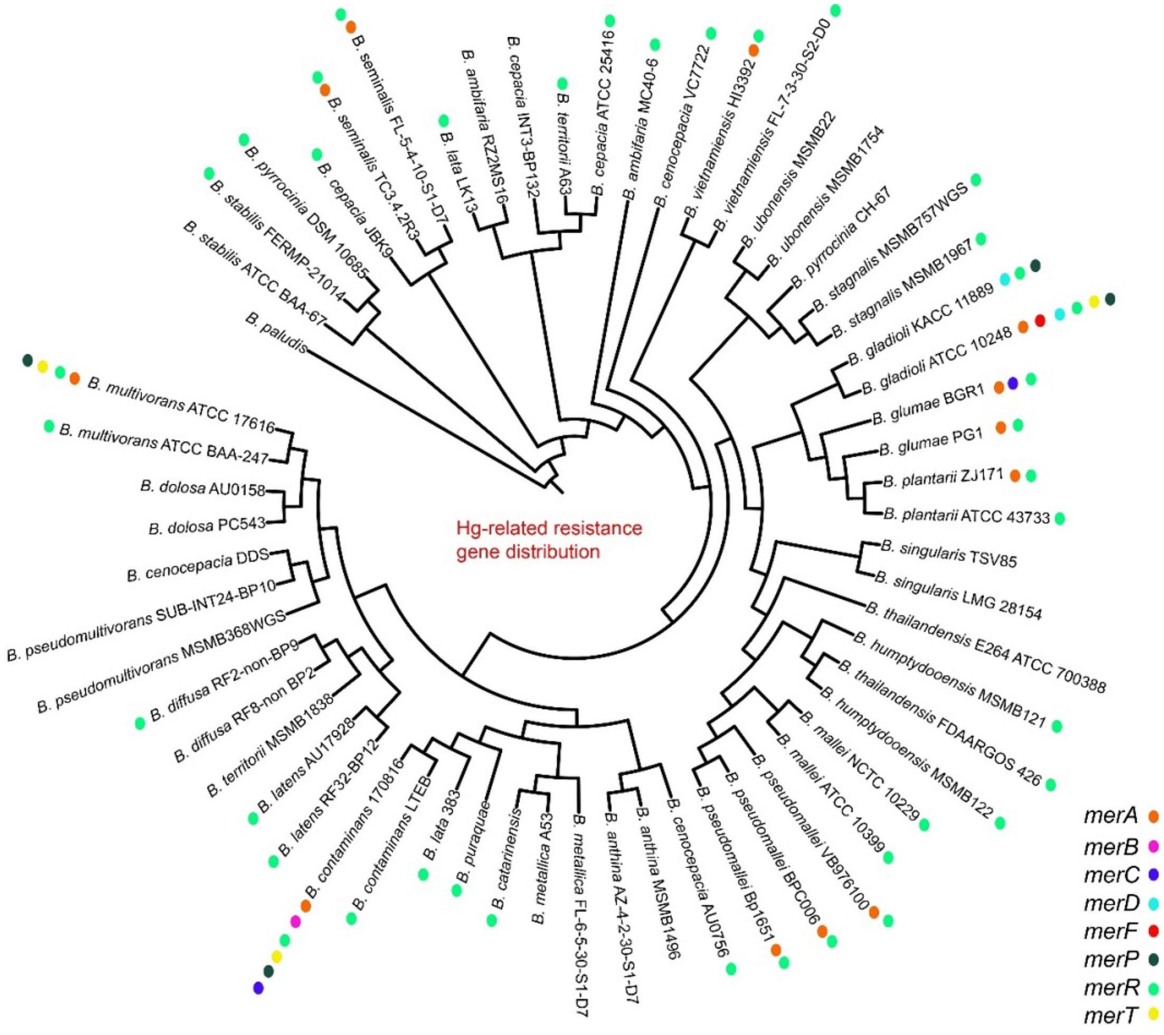
Distribution of mercury resistance genes on 60 *Burkholderia* genomes.

**Figure 6:**
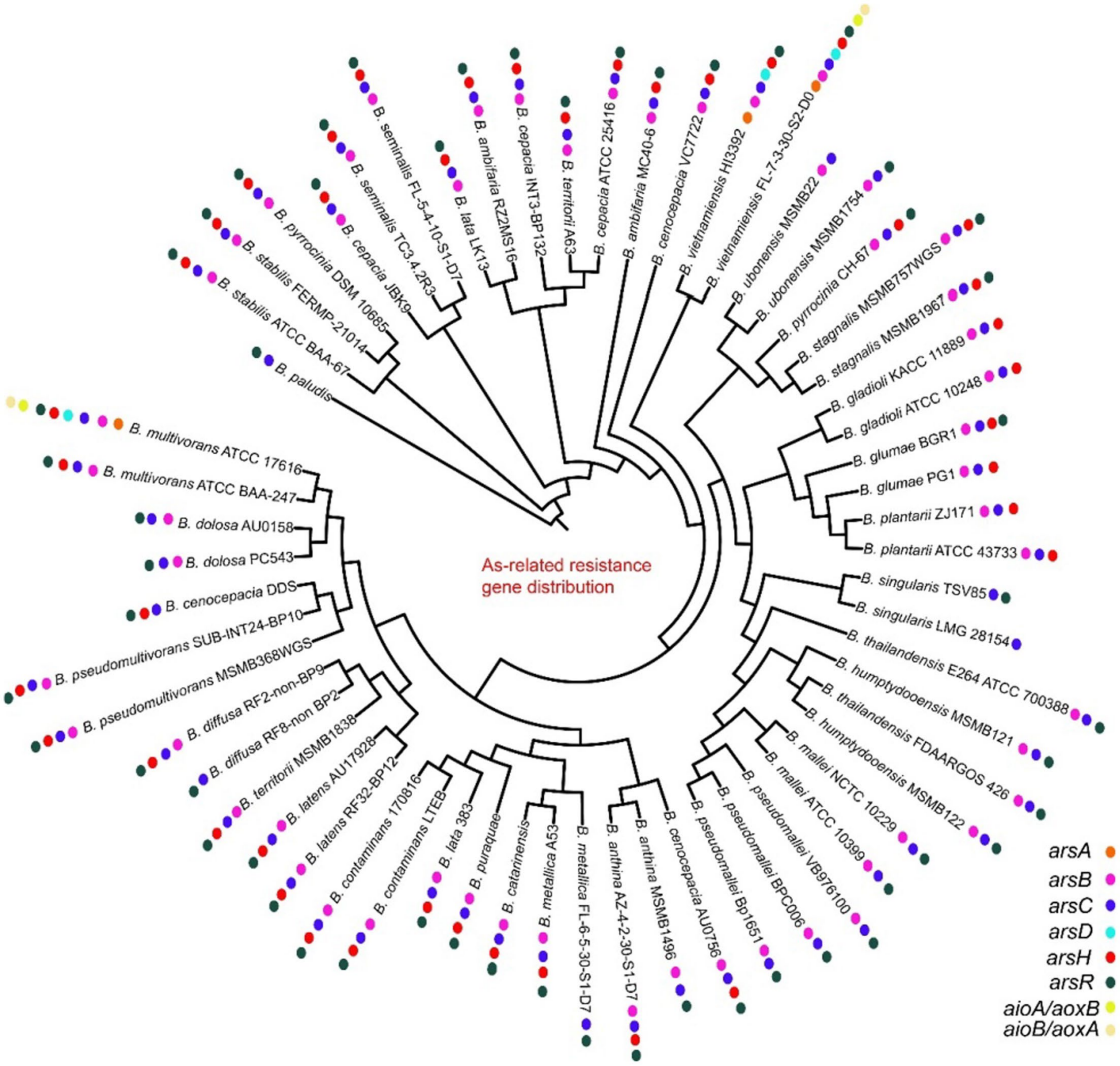
Distribution of arsenic resistance genes on 60 *Burkholderia* genomes.

**Figure 7:**
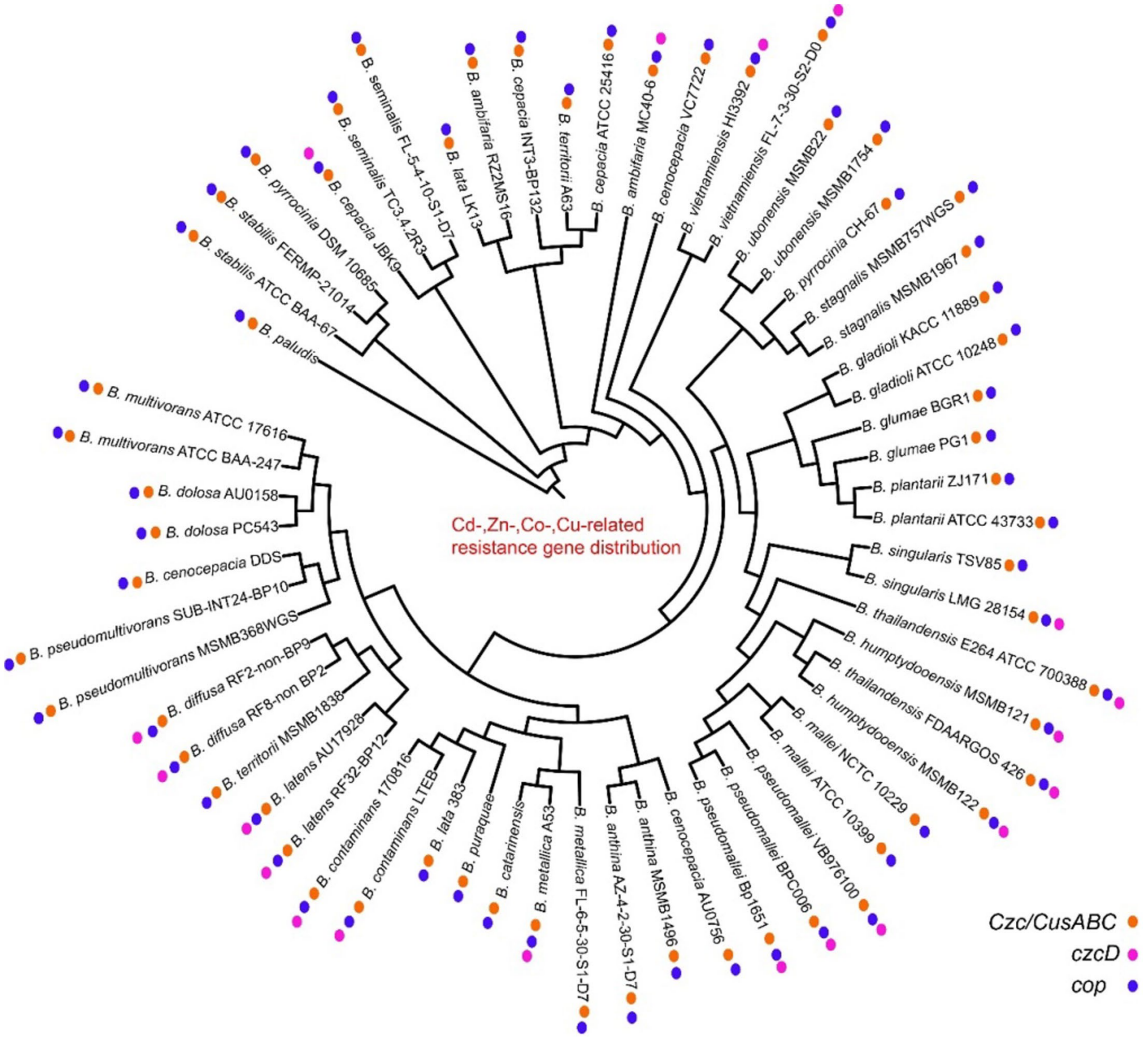
Distribution of cadmium, zinc, cobalt, copper resistance genes on 60 *Burkholderia* genomes.

Heavy metal genes are distributed in most strains, and they are clustered and divided into multiple subgroups. The mercury genes were divided into merA, merR, merG, merRTP, merRTPCAB, merDAFPTR, etc. subgroups, with some strains including two or more mer cluster subgroups (Figure 5; Supplementary Table S4). The arsA and arsD of arsenic and arsenate resistance genes were only identified in some genomes of vietnamiensis (*B. cenocepacia, B. cepacian, B. contaminans, B. gladioli, B. glumae, B. ubonensis, B. vietnamiensis*) (Figure 6; Supplementary Table S5). Aio (A/B) and arr (A/B) were involved in arsenous acid oxidation and arsenate respiration reduction, and aio (A/B) was only identified in *B. centocepacia*, *B. cepacian*, *B. multivorans*, *B. pseudomallei*, *B. ubonensis*, *B. vietnamiensis*, while arr (A/B) was not identified in the *Burkholderia* genus. The distribution of cadmium, zinc, cobalt, and copper resistance genes in the *Burkholderia* genus was shown in Figure 7 and Supplementary Table S6. The czc/cusABC gene cluster was involved in the detoxification of divalent cations (cadmium, zinc and cobalt) and monovalent cations (copper and silver). The genes encoding CzcD and copper translocated P-type ATPase (Cop) had been identified in some *Berkholderia* genomes, most of which were located next to the czc/cusABC gene cluster, while the genes encoding copper oxidase (mco) had not been detected in the *Berkholderia* genome.

The different distributions and combinations of heavy-metal genes indicate differences of origin and evolution. The GC-content analysis of the strains showed that the GC-content of the strains containing large heavy-metal clusters was lower than the genome GC-content of the strains. To further elucidate the evolution of heavy-metal, it was found that upstream and downstream of these large gene clusters contain multiple sequences of transposase, integrase, site-specific recombination enzyme and phage origin, which indicates that the origin of these clusters may be transferred to the genome through horizontal transfer. The analysis of the upstream and downstream clusters of the strain showed that different clusters have different insertion positions in different strains, indicating that the cluster may have been obtained more than once in this genus of species.

**Figure 8:**
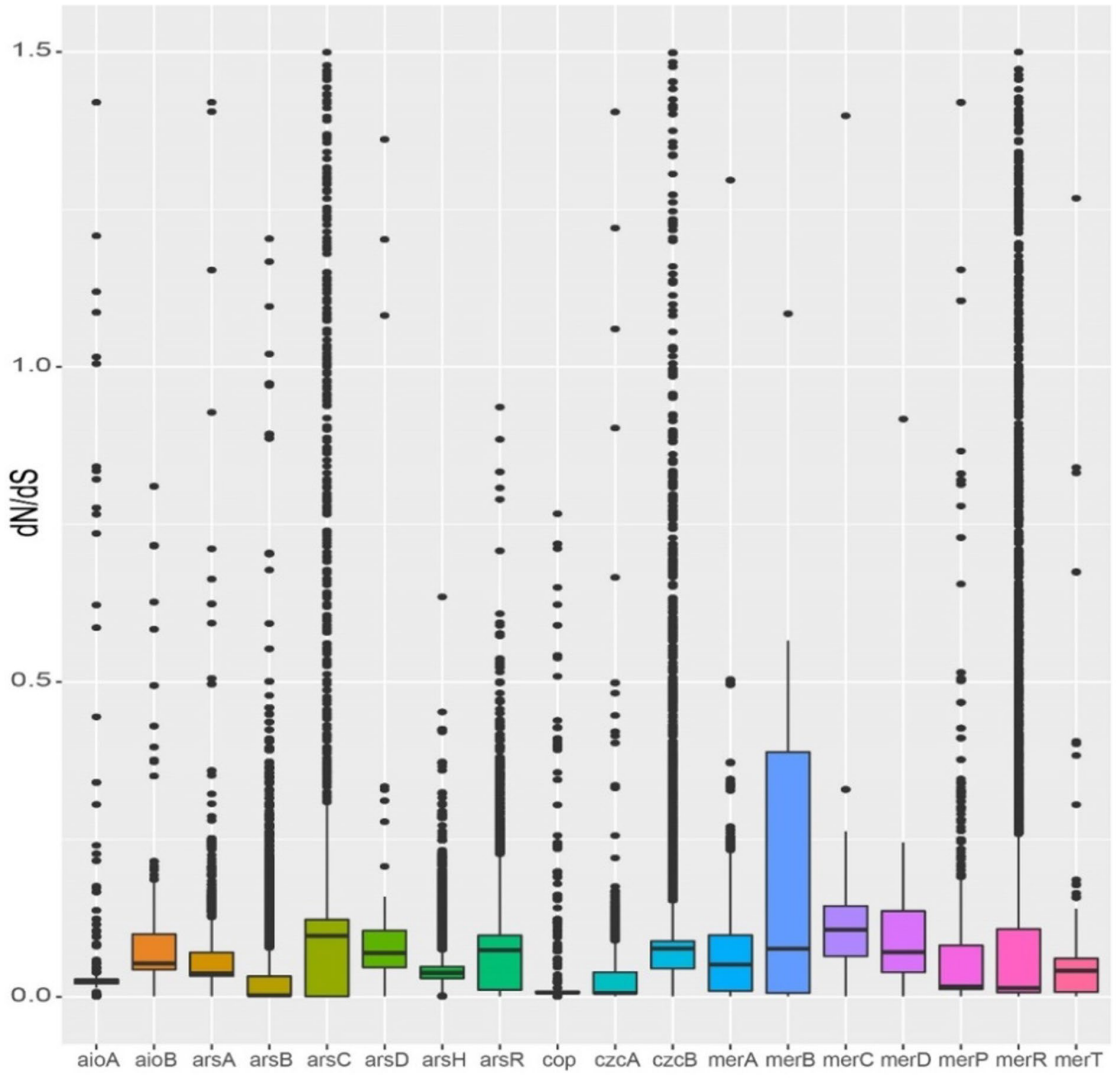
Selection pressures on metal resistance genes of *Burkholderia*.

The dN/dS ratios of almost all heavy metal resistance genes were less than 1, indicating that these genes are subject to purification selection (Figure 8). The dN/dS ratios of arsB, czcB, and merR genes were greater than 1, indicating that of them may be in positive selection. The ArsB (average dN/dS ≤ 0.03) and czcA (average dN/dS ≤ 0.02) were observed to exhibit the lowest dN/dS ratio, indicating strong purification selection (Figure 8).

### Tn7 Like transposable element associated heavy metal resistance genes in *Burkholderia*

**Figure 9:**
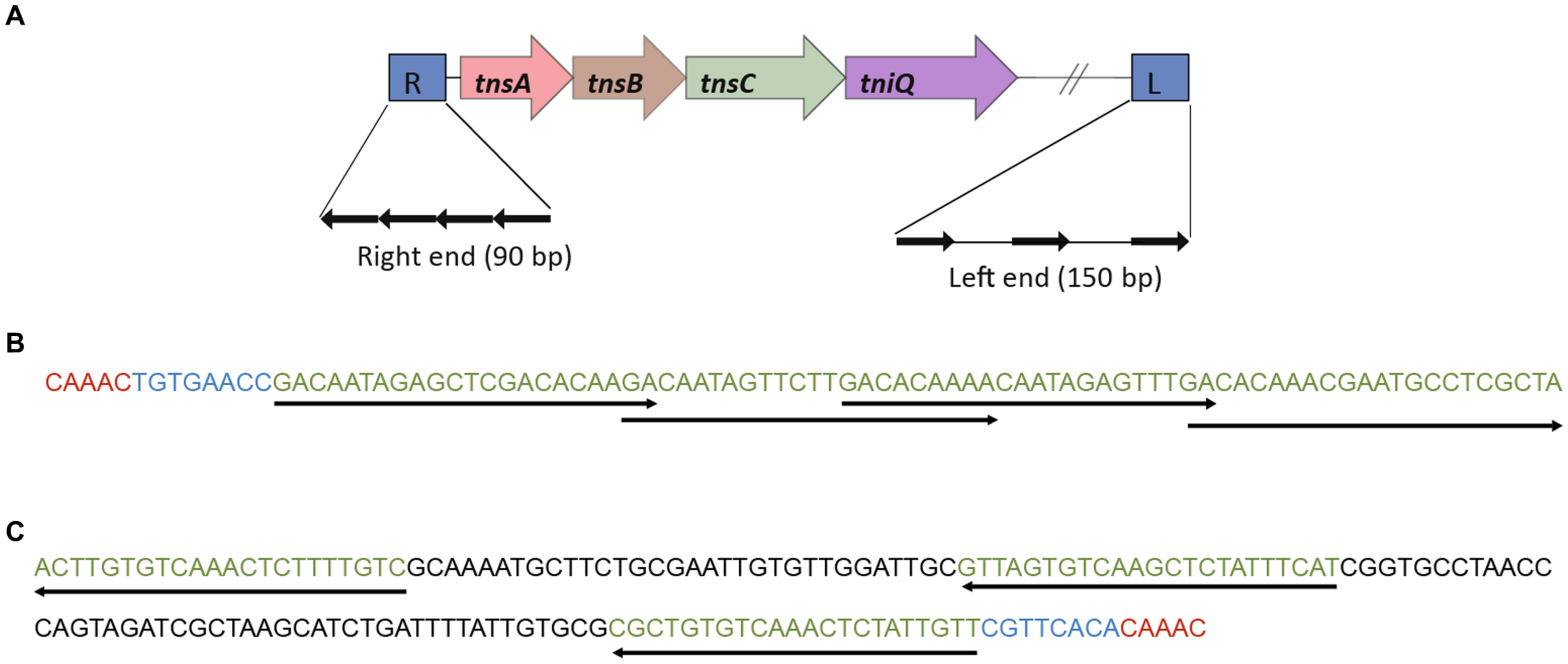
Gene island BGImetal was associated with a Tn7-like structure. **(A)** Tn7-like structure, four genes (*tnsA*, *tnsB*, *tnsC*, *tniQ*) were arranged in sequence on the genome. **(B)** *Burkholderia multivorans* DDS 15A-1 Tn7-like structure right end sequence (90 bp). **(C)** *Burkholderia multivorans*. The left-end sequence (158 bp) of the Tn7-like structure of DDS 15A-1.

**Figure 10:**
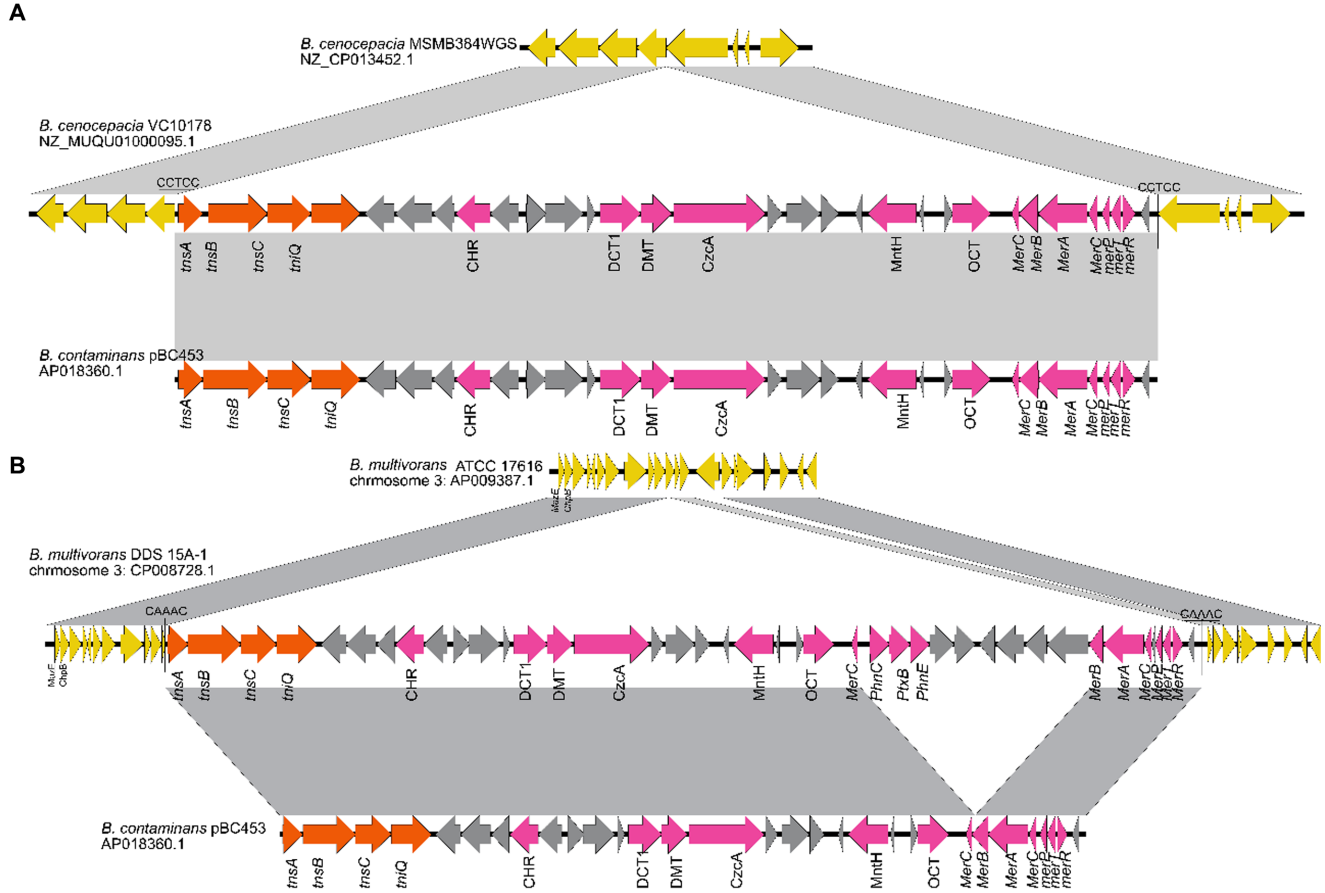
**(A)** *B. cenocepacia* VC10178 genomic fragment has a longer 33,652 bp region than the *B. cenoc* epacia MSMB384WGS chromosome 2, there is also a highly similar fragment in the some *B. cenoc* taminans genome; **(B)** *B. multivoran* DDS 15A-1 chromosome 3 interval compared to *B. multivoran* ATCC 17616 Chromosome 3 has a 43,240 bp region.

**Figure 11:**
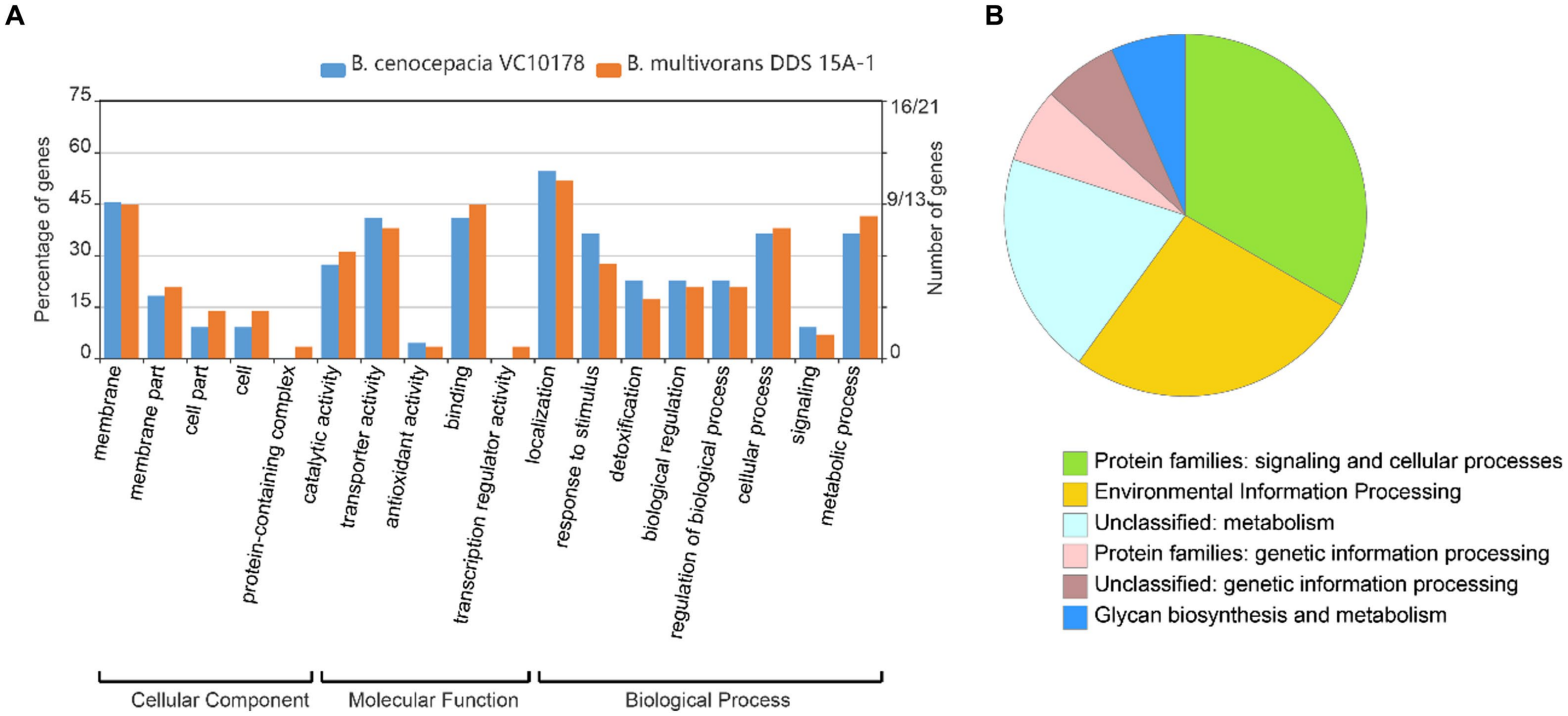
GO, KEGG function annotation gene island BGImetal. **(A)** GO annotation *B. multivoran* DDS 15A-1, *B. cenocepacia* VC10178 gene island BGImetal; **(B)** KEGG annotation *B. multivoran* DDS 15A-1 gene island BGImetal.

1831 genomes were annotated by prokka, domain, and blast, and the results showed that the Tn7 like element is commonly present in the *Burkholderia* genus (Supplementary Figure S3; Supplementary Table S7). The Tn7 like element structures of the three strains (*B. cenocepacia*, *B. multivorans*, *B. contaminans*) contained a special gene island (Figures 9, 10; Supplementary Table S8). The gene island is named ‘BGImetal’. The Open reading frame of BGImetal was annotated using prokka software, the results showed that most ORFs on BGImetal were related to heavy metal resistance. The GO function of the Tn7 like element region was enriched in localization, response to stimuli, detoxification, and biological regulation. KEGG analysis showed that the proteins of this region mainly involve in two pathways: “signaling and cellular processes” and “environmental information processing” (Figures 11A,B).

### Gene island BGImetal was obtained through horizontal transfer

**Figure 12:**
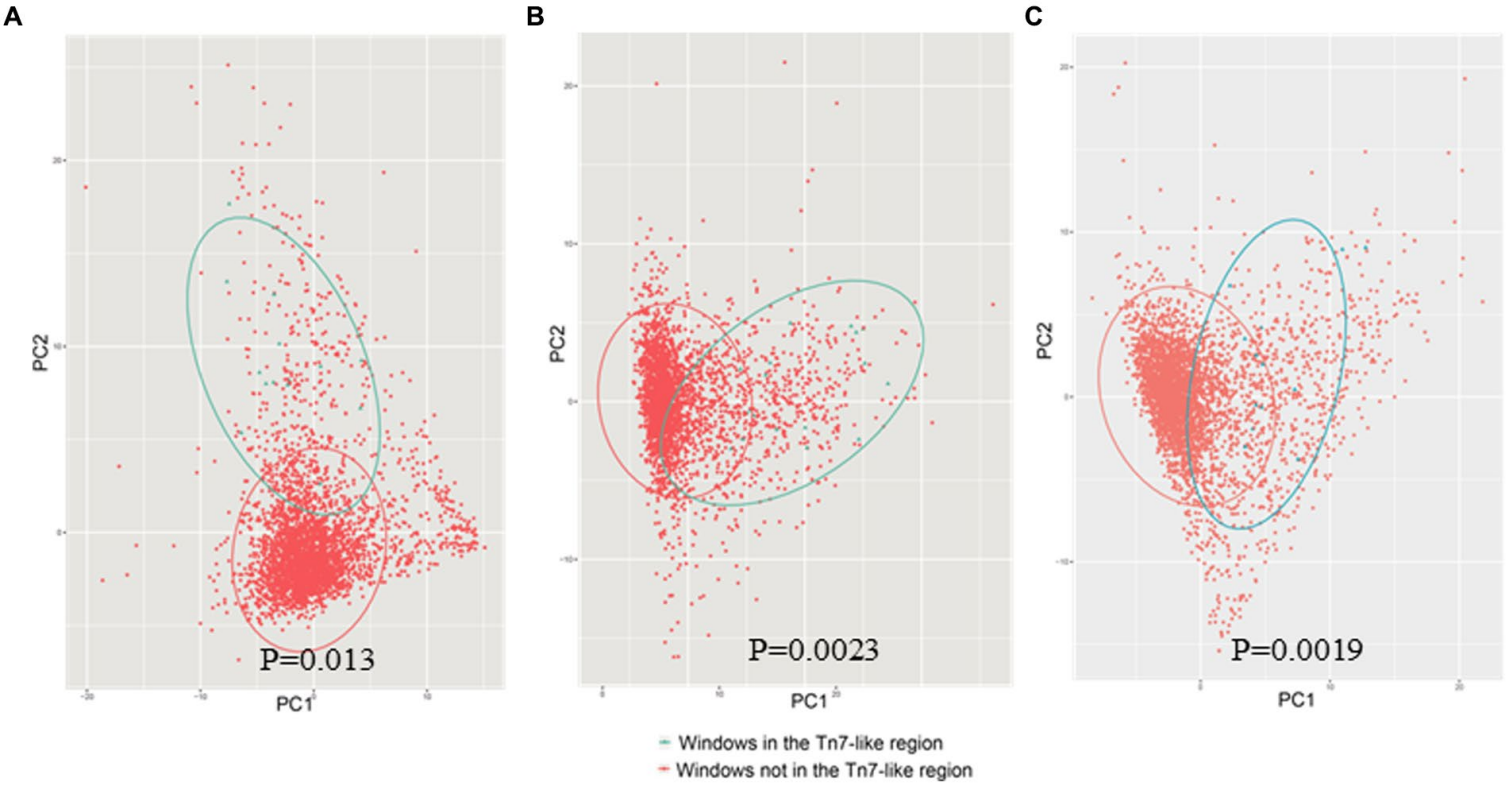
PCA plot of codon usage frequency **(A)** was represented as *B. multivoran* DDS15A-1; **(B)** was represented as *B. cenocepacia* VC10178; **(C)** was represented as *B. cenocepacia* LMG 23361. The blue dots represent the codon usage bias of the gene island BGI metal, and the red dots represent the codon usage bias of other regions on the genome.

The annotation results of the gene island showed that the 574,969–618,208 bp segment of chromosome 3 (CP008728.1) in *B. multiorans* DDS 15A-1 was a Tn7 like element. The GC-content of this region (61.72%) was significantly different from the average GC-content of the whole genome (66.60%). The 23,162–56,803 bp segment of a contig (NZ_MUQU01000095.1) of genome sequence in *B. cenocepacia* VC10178 was a Tn7 like element. The GC-content of this region (62.78%) was significantly different from the average GC-content of the whole genome (67.04%). The GC-content of Tn7 like element region of *B. contaminans* LMG 23361 (62.72%) was different from the average GC-content of the whole genome (65.89%). Sliding window (5 kb size; 2.5 kb step size) was used to statistics the frequency of codon usage in various regions of the *B. multivorans* DDS 15A-1, *B. Centocepacia* VC10178, *B. contraminans* LMG 23361 genome (Figure 12). The principal component analysis indicated that the codon preferences of these two regions are different from the majority of genes. The annotation analysis of gene island indicated that approximately 14.28% of the entire genome of *B. multivorans* DDS 15A-1 is a gene island region, with approximately 25.67% of the region on chromosome 3 (CP008728.1) being a gene island. These results demonstrated that this region is a horizontal gene transfer insertion event into the genome based on Tn7 like elements.

## Discussion

### Classification of *Burkholderia* genus

Once long ago some researchers have only used the method of constructing 16S rRNA (or 16S rDNA) phylogenetic trees to identify the taxonomic status of *Burkholderia* strains (Bazhanov et al., 2010; Schönmann et al., 2010; Sawana et al., 2014). However, there was a high similarity of 16S rRNA among *Burkholderia* strains, and the resolution of 16S rRNA in taxonomy analysis is poor only based on 16S rRNA. It was not reliable to identify strains only through 16S rRNA phylogenetic tree. It was necessary to supplement identification through phylogenetic research of other Housekeeping gene. As a result, some strains in online databases (such as NCBI) were misclassified as species. However, many subsequent studies were based on previous classifications, which may result in bias in the results of studies based on previous classifications. Previous studies had found that the classification of some *Burkholderia* genera was incorrect (Meza-Radilla et al., 2019). The method of constructing phylogenetic trees through the whole genome and core genome to determine the taxonomic status of strains was more reliable than the 16S rDNA method. Therefore, it is recommended that subsequent studies can use the method of constructing phylogenetic trees using whole genome and core genome when identifying the classification of *Burkholderia* species.

In this study, *Pararaburkholderia* was selected as a reference and exogenous species, and compared to other possible options such as the *Ralstonia* genus, *Pararaburkholderia* has a closer genetic relationship with *Burkholderia* (Kaur et al., 2017). Previous studies had shown that using *Pararaburkholderia* as an exogenous species can effectively analyze the main evolutionary branches and phylogenetic relationships of *Burkholderia* (Sawana et al., 2014; Mullins and Mahenthiralingam, 2021). The use of *Pararaburkholderia* will not have a negative impact on phylogenetic of *Burkholderia*. has been added in discussion section. It was found that two bacterial species (*B. dabaoshanensis* and *B. novacaledonica*) were misclassified into the genus *Burkholderia*. The average nucleotide similarity results indicate that *B. dabaoshanensis* and *B. novacaledonica* has a lower average nucleotide similarity (~84%) compared to other strains in the *Burkholderia* genus, while the average nucleotide similarity between strains in the *Burkholderia* genus and *Paraburkholderia phenolitrix* strains in the same family (*Burkholderia ceae*) is 85%, indicating *B. dabaoshanensis* and *B. novacaledonica* may not belong to the *Burkholderia* genus, meaning that these two species belong to *Burkholderia* and are mistakenly defined as genera; The phylogenetic tree results of whole genome construction will also be *B. dabaoshanensis*, *B. novacaledonica* and *P. phenoliruptrix* converge into one branch; Through pan-genome analysis, only *B. dabaoshanensis* and *B. novacaledonica* had a large number of unique genes (1,609 and 1875, respectively), while other strains had fewer than 1,000 unique genes; Based on the above results, it is believed that *B. dabaoshanensis* and *B. novacaledonica* was misclassified into the genus *Burkholderia*.

### Genome evolution of *Burkholderia*

The *Burkholderia* genus includes some plant and animal pathogens as well as important environmental microorganisms (Nisr et al., 2012; Shen et al., 2014; Artemeva et al., 2021). Due to some *Burkholderia* are clinical pathogenic bacteria and have relatively high antibiotic resistance (Arora et al., 2021; Peng et al., 2021; Schaumburg et al., 2022), they can be used for biodegradation, prevention of various plant diseases, production of enzymes, and remediation of heavy metals in soil (Park, 2012; Vu and Moreau, 2017; Liu et al., 2021), which has attracted widespread attention from researchers, and new strains continue to be discovered.

It was found that *Burkholderia* species have rapidly differentiated (Shion et al., 2016). The divergent time tree constructed by pan-genome analysis showed that *Burkholderia* has differentiated into several species at 0 ~ 0.05 MYA, and differentiated strains such as *B. humptydooensis*, which was classified and identified in 2017, belongs to the *Burkholderia pseudomallei* complex. Other recently differentiated species include *B. pseudomallei, B. mallei*, and *B. thailandiensis* et al. Pan-genome analysis showed that the number of core genomes is 1,368 (28.6% of the genes in each strain were core genes on average), and the specific genes of each strain ranged from 46 to 797 (the average number of specific genes of each strain was 242). The unique genes of bacterial strains were usually considered as newly generated genes during the evolutionary process of bacteria, and horizontal gene transfer is the main driving force for bacteria to obtain new genes, which indicating that the *Burkholderia* genus has a strong ability to obtain genes from the outside world.

There were numerous transposable events on the genome (Bishop and Rachwal, 2014). The analysis of codon usage frequency showed that a significant portion of the genome of *Burkholderia* has different codon preferences compared to most regions on the genome. The analysis results of gene islands show that there is a certain proportion of gene island regions in the genome of the *Burkholderia*, such as, approximately 14.28% of the genome of *B. multivoran* DDS 15A-1 is a gene island region, indicating that the *Burkholderia* genus obtains genes from the outside world through horizontal transfer and other methods to enrich its own genome. Based on the above results, it was believed that the *Burkholderia* genus has a faster evolutionary rate. Due to the widespread impact of the *Burkholderia* genus on humans, animals, and plants, as well as the rapid evolution rate of the *Burkholderia* genus, it was possible to isolate and identify more members of the genus in more habitats in the future, and to discover more life and production applications of *Burkholderia* bacteria in the future.

### Acquisition of heavy metal resistance genes in *Burkholderia*

In recent years, many new strains of *Burkholderia* had been tested and analyzed, and it was found that some *Burkholderia* have high efficiency in biological pest control and bioremediation, including resistance to heavy metals (Nguyen et al., 2021; Simonetti et al., 2021). Experimental studies had shown that the *Burkholderia* genus is resistant to various heavy metals such as mercury, lead, cadmium, manganese, etc. (Min et al., 2013; Si et al., 2017; Wang et al., 2020). This study explored the resistance of *Burkholderia* to some heavy metals at the genomic level. It was found that heavy metal resistance genes appeared in clusters in *Burkholderia*, and there were differences in heavy metal resistance genes in different strains. The observation of the upstream and downstream of heavy metal clusters found that there were transposase, recombinant enzyme, integrase and other genes, indicating that these genes may be obtained through horizontal transfer. *B. centocepacia*, *B. multivorans*, B. genes related to resistance to heavy metals such as mercury and manganese were found on the gene island BGImetal in the three strains of contaminans. The gene island BGImetal was horizontally transferred through Tn7 Transposable element in these three strains (Figures 9, 10). The gene island BGImetal associated with Tn7 like was a form of heavy metal resistance gene transfer in the genus *Burkholderia*. The horizontal transfer of other heavy metal resistance genes (or clusters) in *Burkholderia* may take the form of other transposable element. With the increasing severity of heavy metal pollution, pollution control has become a focus of attention. At present, heavy metal resistance genes have been identified in other bacterial genus. Yang et al. studies results found that two kinds of copper-tolerant bacteria and copper-tolerant yeast can with stand Cu2^+^. The cadmium-tolerant Cellulosimicrobium sp. strain and Enterococcus sp.stain can with stand of Cd2^+^. Lead-resistant bacteria of the Lysinibacillus sp. stain and the cadmium-resistant Microbacterium sp. strain can with stand Pb2^+^ (Yang, 2020). *Pesudomonas aeruginosa* M1 has the ability to resist cadmium The cadmium resistant fungal strain janthinellum Penicillium ZZ-2 has the potential to improve the growth, cadmium accumulation, and cadmium tolerance of dog tooth root plants (Xie et al., 2021). Whether the metal resistance genes of these bacterial genus are located on the genome island has not been reported yet.

## Conclusion

1841 genomes of the *Burkholderia* genus were obtained from the NCBI database and filtered by BUSCO to obtain 1831 genomes and 32 bacterial species. The Pan-genome analysis of 62 genomes showed that *B. dabaoshanensis* and *B. novacaledonica* has an abnormal number of unique genes compared to other genomes. The whole genome phylogenetic tree would be *B. dabaoshanensis*, *B. novacaledonica* and *P. phenolitrix* converge to form a branch, indicating *B. dabaoshanensis* and *B. novacaledonica* did not belong to the *Burkholderia* genus. Removing *B. dabaoshanensis* and *B. novacaledonica* genome, pan-genome analysis showed that many core genes were involved in heavy metal resistance. By analyzing the distribution and characteristics of heavy metals such as mercury, arsenic, cadmium, zinc, cobalt and copper in the genus *Burkholderia*, it was found that heavy metal resistance genes appeared in clusters in the genus *Burkholderia*. At the same time, the GC-content of the heavy metal cluster was different from that of the genome, and there were transposase, recombinase, integrase and other genes in the upstream and downstream of the heavy metal cluster, which might be obtained through horizontal transfer. The dN/dS ratio of heavy metal resistance genes in *Burkholderia* was less than 1, indicating that the heavy metal resistance gene undergoes purification selection in the *Burkholderia* genus, and also showing that the heavy metal resistance gene is crucial for the growth of the *Burkholderia* genus.

Tn7 like transposable element was identified in *Burkholderia*. It was found that Tn7 like transposable element are ubiquitous in *Burkholderia*, and they were inserted in various positions in the genome. Due to the evolution of the genome, some Tn7-like transposable element were no longer complete (some Tn7 proteins are lost, and the protein structure is incomplete). It was found that there is a special Tn7 like transposable element that has formed a gene island form (BGI metal) in the genus *Burkholderia*, and this transposable element was only found in *B. Centocepacia*, *B. multivorans*, *Burkholderia contaminans*. The difference of GC-content and codon usage frequency between BGImetal region and other regions of the genome showed that the gene island is horizontally transferred through Tn7 transposable element. Observing the Tn7 element in the structure of BGImetal, it was found that the structure and sequence of Tn7 proteins (TnsA, TnsB, TnsC, TnsD/TniQ) have not undergone a certain degree of change, indicating that the gene island has recently been horizontally transferred into the genome, and the Tn7 protein, L, and R end sequences of the Tn7 element in all BGImetal structures are completely consistent. It was speculated that all discovered BGImetal are from the same source. Meanwhile, the discovery of this structure in all three bacterial species indicates that the gene island has already spread horizontally within the genus. The heavy metal resistance gene carried by Tn7 transposable element in *Burkholderia* was beneficial to the host, making the host become a dominant strain of heavy metal resistance, thus the host has the ability to survive in the environment polluted by heavy metals. Tn7 structure was retained because it is beneficial to the host.

## Data availability statement

The original contributions presented in the study are included in the article/Supplementary material, further inquiries can be directed to the corresponding authors.

## Author contributions

YL, ML, and YC: conceptualization. YL, YS, and FL: investigation. YL, YS, and DL: visualization. YL: writing – original draft. YL, DQ, and DL: writing – review editing. All authors contributed to the article and approved the submitted version.

## Footnotes

^1^https://www.ncbi.nlm.nih.gov/

^2^www.ub.edu/dnasp
