# Supplementary material for: Distribution, characterization, and evolution of heavy metal resistance genes and Tn7-like associated heavy metal resistance Gene Island of Burkholderia
Source: Front Microbiol. 2023 Nov 23;14:1252127. doi: 10.3389/fmicb.2023.1252127 (PMC10702557; doi:10.3389/fmicb.2023.1252127)
Supplement: Supplementary file 1 [file Data_Sheet_1.docx]

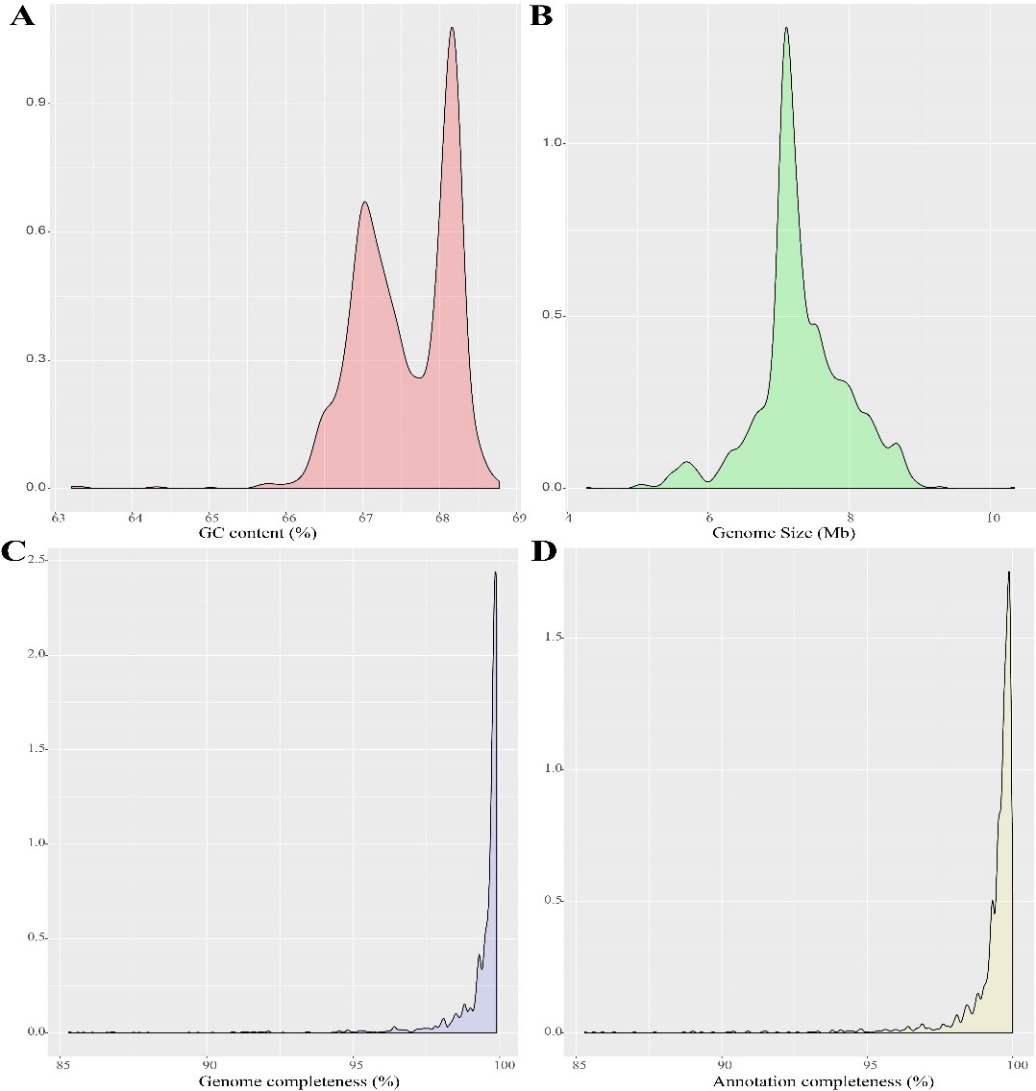


**Figure S1 Genomic features *Burkholderia*. A) GC content nuclear density map; B) genome size nuclear density map; C) BUSCO assessed genome integrity nuclear density map; D) BUSCO assessed proteomic integrity nuclear density map.**

**Table 1. The strain used in the experiment. The number of original genomes not downloaded in parentheses, outside the brackets is the number of genes remaining after BUSCO assessment. * indicates that the strain belongs to Bcc.**

| Species | Total number of strains | type strains | Total number of plasmids |
| --- | --- | --- | --- |
| *B. pseudomallei* | 739（741） | *B. pseudomallei* Mahidol-1106a | 10 |
| *B. cenocepacia** | 258（262） | *B. cenocepacia* MSMB384WGS | 16 |
| *B. ubonensis** | 294 | *B. ubonensis* ATCC 31433 | 6 |
| *B. cepacia** | 97 | *B. cepacia* BC16 | 30 |
| *B. multivorans** | 88（90） | *B. multivorans* FDAARGOS_246 | 8 |
| *B. mallei* | 64 | *B. mallei* Turkey2 | 10 |
| *B. stagnalis** | 64 | *B. stagnalis* MSMB735WGS | 0 |
| *B. vietnamiensis** | 43 | *B. vietnamiensis* FL-2-3-10-S3-D0 | 11 |
| *B. thailandensis* | 42 | *B. thailandensis* 2002721643 | 4 |
| *B. territorii** | 34 | *B. territorii* RF8-non-BP5 | 0 |
| *B. gladioli* | 19 | *B. gladioli* BBB-01 | 30 |
| *B. diffusa** | 12 | *B. diffusa* LMG 29043 | 0 |
| *B. contaminans** | 10 | *B. contaminans* CH-1 | 1 |
| *B. ambifaria** | 9 | *B. ambifaria* FDAARGOS_1027 | 10 |
| *B. pseudomultivorans** | 9 | *B. pseudomultivorans* BCC1191 | 2 |
| *B. anthina** | 8 | *B. anthina* LMG 20980 | 0 |
| *B. glumae* | 8（9） | *B. glumae* 257sh-1 | 15 |
| *B. pyrrocinia** | 5 | *B. pyrrocinia* DSM 10685 | 2 |
| *B. lata** | 4 | *B. lata* FDAARGOS_899 | 0 |
| *B. humptydooensis* | 3 | *B. humptydooensis* FDAARGOS_899 | 0 |
| *B. seminalis** | 3 | *B. seminalis* FL-5-4-10-S1-D7 | 0 |
| *B. stabilis** | 3 | *B. stabilis* E | 2 |
| *B. dolosa** | 3 | *B. dolosa* AU0158 | 0 |
| *B. latens** | 2 | *B. latens* BCC1625 | 2 |
| *B. metallica** | 2 | *B. metallica* FL-6-5-30-S1-D7 | 0 |
| *B. plantarii* | 2 | *B. plantarii* ATCC 43733 | 2 |
| *B. singularis* | 2 | *B. singularis* TSV85 | 0 |
| *B. catarinensis** | 1 | *B. catarinensis* 89 | 0 |
| *B. dabaoshanensis* | 1 | *B. dabaoshanensis* GIMN1.004 | 0 |
| *B. paludis** | 1 | *B. paludis* LMG 30113 | 0 |
| *B. puraquae** | 1 | *B. puraquae* LMG 29660 | 1 |
| *B. novacaledonica* | 1 | *B. novacaledonica* LMG 28615 | 0 |
| *Burkholderia* *sp.* | 0 |  | 77 |

**Table S2 General features of bacterial genomes used in pan-genome analysis**

| Species | Strain | GenBank no. | Level | Size(Mb) | GC(%) | No. of genes | No. of protein |
| --- | --- | --- | --- | --- | --- | --- | --- |
| *B. pseudomallei* | BPC006 | GCA_000294635.1 | Complete | 7.16 | 68.22 | 7373 | 6885 |
| *B. pseudomallei* | Bp1651 | GCA_001318245.1 | Complete | 7.26 | 68.10 | 7499 | 6817 |
| *B. pseudomallei* | VB976100 | GCA_001885195.1 | Complete | 7.28 | 68.02 | 7507 | 6865 |
| *B. cenocepacia* | DDS 22E-1 | GCA_000755725.1 | Complete | 8.05 | 66.97 | 7347 | 7030 |
| *B. cenocepacia* | VC7722 | GCA_001992875.1 | Contig | 8.15 | 66.90 | 7926 | 7352 |
| *B. ubonensis* | A21 | GCA_001957105.1 | Scaffold | 7.01 | 67.30 | 6390 | 6064 |
| *B. ubonensis* | MSMB22 | GCA_000959245.1 | Complete | 7.19 | 67.31 | 6473 | 6127 |
| *B. ubonensis* | MSMB1754 | GCA_001527465.1 | Contig | 7.60 | 67.30 | 6912 | 6573 |
| *B. cepacia* | ATCC_25416 | GCA_001411495.1 | Complete | 8.61 | 66.60 | 8014 | 7614 |
| *B. cepacia* | INT3-BP132 | GCA_001525965.1 | Contig | 8.74 | 66.50 | 8141 | 7690 |
| *B. cepacia* | JBK9 | GCA_000701165.2 | Complete | 8.48 | 66.81 | 7822 | 7476 |
| *B. multivorans* | ATCC 17616 | GCA_000018505.1 | Complete | 7.01 | 66.69 | 6739 | 6397 |
| *B. multivorans* | ATCC BAA-247 | GCA_000959525.1 | Complete | 6.32 | 67.20 | 6016 | 5787 |
| *B. mallei* | ATCC 10399 | GCA_000152305.1 | Scaffold | 5.93 | 68.40 | 6240 | 5783 |
| *B. mallei* | NCTC 10229 | GCA_000015605.1 | Complete | 5.74 | 68.48 | 6004 | 5602 |
| *B. stagnalis* | MSMB1967 | GCA_001527725.1 | Contig | 7.69 | 67.60 | 7004 | 6642 |
| *B. stagnalis* | MSMB757WGS | GCA_001534205.1 | Contig | 7.51 | 67.70 | 6852 | 6503 |
| *B. vietnamiensis* | FL-7-3-30-S2-D0 | GCA_001524225.1 | Contig | 6.53 | 67.30 | 5910 | 5641 |
| *B. vietnamiensis* | HI3392 | GCA_001529645.1 | Contig | 7.62 | 66.40 | 7174 | 6781 |
| *B. thailandensis* | E264_ATCC_700388 | GCF_000152285.1 | Complete | 6.35 | 67.63 | 5990 | 5374 |
| *B. thailandensis* | FDAARGOS_426 | GCA_002386205.1 | Complete | 6.73 | 67.47 | 6423 | 5808 |
| *B. territorii* | A63 | GCA_002151255.1 | Scaffold | 8.87 | 66.40 | 8468 | 8047 |
| *B. territorii* | MSMB1838 | GCA_001526425.1 | Contig | 6.82 | 66.80 | 6205 | 5928 |
| *B. gladioli* | ATCC_10248 | GCA_000959725.1 | Complete | 8.90 | 67.62 | 7729 | 7386 |
| *B. gladioli* | FDAARGOS_389 | GCA_002393425.1 | Complete | 8.29 | 67.97 | 7271 | 6978 |
| *B. diffusa* | RF8-non_BP2 | GCA_001522375.1 | Contig | 5.84 | 66.70 | 5410 | 5173 |
| *B. diffusa* | RF2-non-BP9 | GCA_001718315.1 | Complete | 6.86 | 66.46 | 6346 | 6059 |
| *B. contaminans* | 170816 | GCA_002924455.1 | Complete | 8.63 | 66.46 | 8127 | 7632 |
| *B. contaminans* | LTEB | GCA_001865715.1 | Contig | 8.00 | 66.50 | 7394 | 7075 |
| *B. ambifaria* | RZ2MS16 | GCA_001443045.1 | Scaffold | 8.10 | 65.70 | 7551 | 7000 |
| *B. ambifaria* | MC40-6 | GCA_000019925.1 | Complete | 7.64 | 66.38 | 6739 | 6575 |
| *B. pseudomultivorans* | MSMB368WGS | GCA_001532035.1 | Contig | 7.74 | 66.90 | 7162 | 6793 |
| *B. pseudomultivorans* | SUB-INT24-BP10 | GCA_001525825.1 | Contig | 7.91 | 67.40 | 7244 | 6939 |
| *B. anthina* | MSMB1496 | GCA_001527785.1 | Contig | 7.51 | 67.00 | 6974 | 6595 |
| *B. anthina* | AZ-4-2-30-S1-D7 | GCA_001523905.1 | Contig | 7.20 | 67.20 | 6703 | 6325 |
| *B. glumae* | PG1 | GCA_000835205.1 | Complete | 7.90 | 68.78 | 6718 | 6402 |
| *B. glumae* | BGR1 | GCA_000022645.2 | Complete | 7.28 | 67.93 | 6485 | 6151 |
| *B. pyrrocinia* | CH-67 | GCA_000297475.1 | Contig | 8.05 | 67.40 | 7476 | 6945 |
| *B. pyrrocinia* | DSM_10685 | GCA_001028665.1 | Complete | 7.96 | 66.47 | 7298 | 6929 |
| *B. lata* | lata_383 | GCA_000012945.1 | Complete | 8.68 | 66.26 | 7991 | 7678 |
| *B. lata* | lata_LK13 | GCA_001038835.1 | Contig | 8.67 | 66.50 | 8015 | 7599 |
| *B. humptydooensis* | MSMB121 | GCA_002888035.1 | Contig | 6.65 | 67.50 | 6479 | 5732 |
| *B. humptydooensis* | MSMB122 | GCA_001462435.1 | Complete | 6.79 | 67.47 | 6471 | 5891 |
| *B. seminalis* | FL-5-4-10-S1-D7 | GCA_001718535.1 | Complete | 7.65 | 67.27 | 7091 | 6765 |
| *B. seminalis* | TC3.4.2R3 | GCA_001742345.1 | Contig | 7.67 | 67.20 | 7164 | 6799 |
| *B. stabilis* | ATCC_BAA-67 | GCA_001742165.1 | Complete | 8.53 | 66.41 | 7946 | 7510 |
| *B. stabilis* | FERMP-21014 | GCA_002356275.1 | Complete | 7.72 | 66.69 | 7109 | 6770 |
| *B. dolosa* | AU0158 | GCA_000152585.1 | Scaffold | 6.42 | 66.10 | 5058 | 4822 |
| *B. dolosa* | PC543 | GCA_000497165.1 | Complete | 6.30 | 67.05 | 5817 | 5448 |
| *B. latens* | AU17928 | GCA_001718795.1 | Complete | 6.61 | 66.39 | 6145 | 5820 |
| *B. latens* | RF32-BP12 | GCA_001525985.1 | Contig | 6.50 | 66.50 | 5972 | 5689 |
| *B. metallica* | A53 | GCA_003034145.1 | Scaffold | 8.23 | 66.60 | 7848 | 7188 |
| *B. metallica* | FL-6-5-30-S1-D7 | GCA_001718555.1 | Complete | 7.42 | 67.12 | 6827 | 6491 |
| *B. plantarii* | ATCC_43733 | GCA_001411805.1 | Complete | 8.08 | 68.58 | 6879 | 6575 |
| *B. plantarii* | ZJ171 | GCA_001755835.1 | Scaffold | 8.02 | 68.50 | 6920 | 6495 |
| *B. singularis* | LMG_28154 | GCA_900176645.1 | Contig | 5.54 | 64.30 | 4972 | 4653 |
| *B. singularis* | TSV85 | GCA_001523725.1 | Contig | 5.56 | 64.30 | 4909 | 4632 |
| *B. catarinensis* | - | GCA_001883705.1 | Contig | 8.14 | 66.50 | 7709 | 6128 |
| *B. dabaoshanensis* | - | GCA_002879885.1 | Contig | 7.09 | 63.30 | 6514 | 6329 |
| *B. paludis* | - | GCA_000732615.1 | Contig | 8.63 | 67.10 | 7098 | 6963 |
| *B. puraquae* |  | GCA_002099195.1 | Contig | 8.10 | 66.60 | 7574 | 7182 |
| *B. novacaledonica* | - | GCA_900258035.1 | Contig | 6.78 | 63.30 | 6240 | 6179 |

**Table S3 Average nucleotide identity of bacterial genomes used in pan-genome analysis.**

| Species | Strain | GenBank no. | Level | Size(Mb) | GC(%) | No. of genes | No. of protein |
| --- | --- | --- | --- | --- | --- | --- | --- |
| *B. pseudomallei* |  |  |  |  |  |  |  |
| *B.* *cenocepacia* | AU0756 | GCF_002981045.1 | Contig | 7.26 | 67.00 | 6853 | 6509 |
| *B. cenocepacia* | DDS 22E-1 | GCF_000755725.1 | Complete | 8.05 | 66.97 | 7347 | 7030 |
| *B. cenocepacia* | VC7722 | GCF_001992875.1 | Contig | 8.15 | 66.9 | 7926 | 7352 |
| *B.* *ubonensis* |  |  |  |  |  |  |  |
| *B.* *cepacia* | ATCC_25416 | GCA_001411495.1 | Complete | 8.61 | 66.60 | 8014 | 7614 |
| *B. cepacia* | INT3-BP132 | GCA_001525965.1 | Contig | 8.74 | 66.50 | 8141 | 7690 |
| *B. cepacia* | JBK9 | GCA_000701165.2 | Complete | 8.48 | 66.81 | 7822 | 7476 |
| *B.* *multivorans* | ATCC 17616 | GCA_000018505.1 | Complete | 7.01 | 66.69 | 6739 | 6397 |
| *B. multivorans* | ATCC BAA-247 | GCA_000959525.1 | Complete | 6.32 | 67.20 | 6016 | 5787 |
| *B.* *mallei* | ATCC 10399 | GCA_000152305.1 | Scaffold | 5.93 | 68.40 | 6240 | 5783 |
| *B. mallei* | NCTC 10229 | GCA_000015605.1 | Complete | 5.74 | 68.48 | 6004 | 5602 |
| *B.* *stagnalis* | MSMB1967 | GCA_001527725.1 | Contig | 7.69 | 67.60 | 7004 | 6642 |
| *B. stagnalis* | MSMB757WGS | GCA_001534205.1 | Contig | 7.51 | 67.70 | 6852 | 6503 |
| *B. vietnamiensis* | FL-7-3-30-S2-D0 | GCA_001524225.1 | Contig | 6.53 | 67.30 | 5910 | 5641 |
| *B. vietnamiensis* | HI3392 | GCA_001529645.1 | Contig | 7.62 | 66.40 | 7174 | 6781 |
| *B. thailandensis* | E264_ATCC_700388 | GCF_000152285.1 | Complete | 6.35 | 67.63 | 5990 | 5374 |
| *B. thailandensis* | FDAARGOS_426 | GCA_002386205.1 | Complete | 6.73 | 67.47 | 6423 | 5808 |
| *B. territorii* | A63 | GCA_002151255.1 | Scaffold | 8.87 | 66.40 | 8468 | 8047 |
| *B. territorii* | MSMB1838 | GCA_001526425.1 | Contig | 6.82 | 66.80 | 6205 | 5928 |
| *B.* *gladioli* | ATCC_10248 | GCA_000959725.1 | Complete | 8.90 | 67.62 | 7729 | 7386 |
| *B. gladioli* | FDAARGOS_389 | GCA_002393425.1 | Complete | 8.29 | 67.97 | 7271 | 6978 |
| *B. diffusa* | RF8-non_BP2 | GCA_001522375.1 | Contig | 5.84 | 66.70 | 5410 | 5173 |
| *B. diffusa* | RF2-non-BP9 | GCA_001718315.1 | Complete | 6.86 | 66.46 | 6346 | 6059 |
| *B. contaminans* | 170816 | GCA_002924455.1 | Complete | 8.63 | 66.46 | 8127 | 7632 |
| *B. contaminans* | LTEB | GCA_001865715.1 | Contig | 8.00 | 66.50 | 7394 | 7075 |
| *B. ambifaria* | RZ2MS16 | GCA_001443045.1 | Scaffold | 8.10 | 65.70 | 7551 | 7000 |
| *B. ambifaria* | MC40-6 | GCA_000019925.1 | Complete | 7.64 | 66.38 | 6739 | 6575 |
| *B. pseudomultivorans* | MSMB368WGS | GCA_001532035.1 | Contig | 7.74 | 66.90 | 7162 | 6793 |
| *B. pseudomultivorans* | SUB-INT24-BP10 | GCA_001525825.1 | Contig | 7.91 | 67.40 | 7244 | 6939 |
| *B. anthina* | MSMB1496 | GCA_001527785.1 | Contig | 7.51 | 67.00 | 6974 | 6595 |
| *B. anthina* | AZ-4-2-30-S1-D7 | GCA_001523905.1 | Contig | 7.20 | 67.20 | 6703 | 6325 |
| *B. glumae* | PG1 | GCA_000835205.1 | Complete | 7.90 | 68.78 | 6718 | 6402 |
| *B. glumae* | BGR1 | GCA_000022645.2 | Complete | 7.28 | 67.93 | 6485 | 6151 |
| *B. pyrrocinia* | CH-67 | GCA_000297475.1 | Contig | 8.05 | 67.40 | 7476 | 6945 |
| *B. pyrrocinia* | DSM_10685 | GCA_001028665.1 | Complete | 7.96 | 66.47 | 7298 | 6929 |
| *B. lata* | lata_383 | GCA_000012945.1 | Complete | 8.68 | 66.26 | 7991 | 7678 |
| *B. lata* | lata_LK13 | GCA_001038835.1 | Contig | 8.67 | 66.50 | 8015 | 7599 |
| *B. humptydooensis* | MSMB121 | GCA_002888035.1 | Contig | 6.65 | 67.50 | 6479 | 5732 |
| *B. humptydooensis* | MSMB122 | GCA_001462435.1 | Complete | 6.79 | 67.47 | 6471 | 5891 |
| *B. seminalis* | FL-5-4-10-S1-D7 | GCA_001718535.1 | Complete | 7.65 | 67.27 | 7091 | 6765 |
| *B. seminalis* | TC3.4.2R3 | GCA_001742345.1 | Contig | 7.67 | 67.20 | 7164 | 6799 |
| *B. stabilis* | ATCC_BAA-67 | GCA_001742165.1 | Complete | 8.53 | 66.41 | 7946 | 7510 |
| *B. stabilis* | FERMP-21014 | GCA_002356275.1 | Complete | 7.72 | 66.69 | 7109 | 6770 |
| *B. dolosa* | AU0158 | GCA_000152585.1 | Scaffold | 6.42 | 66.10 | 5058 | 4822 |
| *B. dolosa* | PC543 | GCA_000497165.1 | Complete | 6.30 | 67.05 | 5817 | 5448 |
| *B. latens* | AU17928 | GCA_001718795.1 | Complete | 6.61 | 66.39 | 6145 | 5820 |
| *B. latens* | RF32-BP12 | GCA_001525985.1 | Contig | 6.50 | 66.50 | 5972 | 5689 |
| *B. metallica* | A53 | GCA_003034145.1 | Scaffold | 8.23 | 66.6 | 7848 | 7188 |
| *B. metallica* | FL-6-5-30-S1-D7 | GCA_001718555.1 | Complete | 7.42 | 67.12 | 6827 | 6491 |
| *B. plantarii* | ATCC_43733 | GCA_001411805.1 | Complete | 8.08 | 68.58 | 6879 | 6575 |
| *B. plantarii* | ZJ171 | GCA_001755835.1 | Scaffold | 8.02 | 68.50 | 6920 | 6495 |
| *B. singularis* | LMG_28154 | GCA_900176645.1 | Contig | 5.54 | 64.3 | 4972 | 4653 |
| *B. singularis* | TSV85 | GCA_001523725.1 | Contig | 5.56 | 64.3 | 4909 | 4632 |
| *B. catarinensis* | - | GCA_001883705.1 | Contig | 8.14 | 66.5 | 7709 | 6128 |
| *B. dabaoshanensis* | - | GCF_002879885.1 | Contig | 70.9 | 63.3 | 6514 | 6329 |
| *B. paludis* | - | GCF_000732615.1 | Contig | 8.63 | 67.1 | 7098 | 6963 |
| *B. puraquae* |  | GCF_002099195.1 | Contig | 8.10 | 66.6 | 7574 | 7182 |
| *B. novacaledonica* | - | GCA_900258035.1 | Contig | 6.78 | 63.3 | 6240 | 6179 |

**Table S4 Distribution of mercury resistance genes in the genus of *Burkholderia.* The value in parentheses is the number of genomes in which the resistance gene is present and the total number of genomes used for analysis.**

| **Hg resistance gene** | **Species** |
| --- | --- |
| *merA* | *B. cenocepacia*(120/258); *B. cepacian*(2/97); *B. contaminans*(5/10)  *B. gladioli* (3/19); *B. glumae*(5/8); *B. multivorans*(11/88)  *B. plantarii*(1/2); *B. pseudomallei*(630/739); *B. thailandensis*(4/442)  *B. vietnamiensis*(6/43) |
| *merB* | *B. cenocepacia*(77/258); *B. contaminans*(5/10); *B. gladioli*(1/19)  *B. multivorans*(5/88); *B. vietnamiensis*(2/43) |
| *merC* | *B. cenocepacia*(17/258); *B. contaminans*(2/10); *B. gladioli*(1/19)  *B. glumae*(4/19); *B. multivorans*(1/88); *B. thailandensis*(4/442)  *B. vietnamiensis*(2/43) |
| *merD* | *B. cenocepacia*(40/258); *B. contaminans*(1/10); *B. gladioli*(4/19)  *B. multivorans*(2/88); *B. vietnamiensis*(2/43) |
| *merF* | *B. gladioli*(2/19); *B. multivorans*(1/88) |
| *merP* | *B. cenocepacia*(116/258); *B. cepacian*(2/97); *B. contaminans*(5/10)  *B. gladioli*(4/19); *B. humptydooensis*(3/3); *B. multivorans*(11/88)  *B. thailandensis*(39/442); *B. ubonensis*(1/294); *B. vietnamiensis*(4/43) |
| *merT* | *B. cenocepacia*(119/258); *B. cepacian*(2/97); *B. contaminans*(5/10)  *B. gladioli*(3/19); *B. multivorans*(11/88); *B. thailandensis*(4/442)  *B. ubonensis*(1/294); *B. vietnamiensis*(4/43) |

**Table S5 Distribution of arsenic resistance genes in the genus of *Burkholderia*. The value in parentheses is the number of genomes in which the resistance gene is present and the total number of genomes used for analysis.**

| **As resistance gene** | **Species** |
| --- | --- |
| *arsA* | *B. cenocepacia*(6/258); *B. cepacian*(1/97); *B. contaminans*(2/10)  *B. gladioli* (1/19);  *B. glumae*(7/8); *B. ubonensis*(1/294)  *B. vietnamiensis*(440/43) |
| *arsB* | *B. ambifaria*(9/9); *B. anthina*(8/8); *B. cenocepacia*(248/258)  *B. cepacian*(96/97); *B. contaminans*(10/10); *B. diffusa*(11/12)  *B. dolosa*(3/3); *B. gladioli*(19/19); *B. glumae*(2/8); *B. humptydooensis*(3/3)  *B. lata*(4/4); *B. latens*(2/2); *B. mallei*(45/64); *B. metallica*(1/2)  *B. multivorans*(88/88); *B. plantarii*(2/2); *B. pseudomallei*(728/739)  *B. pseudomultivorans*(9/9); *B. puraquae*(1/1); *B. pyrrocinia*(5/5)  *B. seminalis*(3/3); *B. stabilis*(3/3); *B. stagnalis*(64/64)  *B. territorii*(33/34); *B. thailandensis*(42/42); *B. ubonensis*(294/294)  *B. vietnamiensis*(43/43) |
| *arsC* | *B. ambifaria*(9/9); *B. anthina*(8/8); *B. cenocepacia*(258/258)  *B. cepacian*(97/97); *B. contaminans*(10/10); *B. diffusa*(12/12)  *B. dolosa*(3/3); *B. gladioli*(19/19); *B. glumae*(8/8); *B. humptydooensis*(3/3)  *B. lata*(4/4); *B. latens*(2/2); *B. mallei*(63/64); *B. metallica*(2/2)  *B. multivorans*(88/88); *B. novacaledonica*(1/1); *B. paludis*(1/1)  *B. plantarii*(2/2);*B. pseudomallei*(739/739); *B. pseudomultivorans*(9/9)  *B. puraquae*(1/1); *B. pyrrocinia*(5/5);*B. seminalis*(3/3); *B. singularis*(2/2); *B. stabilis*(3/3); *B. stagnalis*(64/64);*B. territorii*(34/34)  *B. thailandensis*(42/42); *B. ubonensis*(294/294);*B. vietnamiensis*(43/43) |
| *arsD* | *B. cenocepacia*(6/258); *B. cepacian*(1/97); *B. contaminans*(2/10);  *B. multivorans*(7/88); *B. vietnamiensis*(14/43) |
| *arsH* | *B. ambifaria*(9/9); *B. anthina*(8/8); *B. cenocepacia*(248/258)  *B. cepacian*(92/97); *B. contaminans*(10/10); *B. diffusa*(11/12)  *B. gladioli*(19/19); *B. glumae*(2/8); *B. lata*(4/4); *B. latens*(2/2)  *B. metallica*(1/2); *B. multivorans*(26/88);*B. paludis*(1/1)  *B. plantarii*(2/2);*B. pseudomallei*(8/739);*B. pseudomultivorans*(9/9)  *B. puraquae*(1/1); *B. pyrrocinia*(5/5); *B. seminalis*(3/3); *B. stabilis*(3/3)  *B. stagnalis*(64/64); *B. territorii*(34/34); *B. ubonensis*(40/294)  *B. vietnamiensis*(19/43) |
| *arsR* | *B. ambifaria*(9/9); *B. anthina*(8/8); *B. cenocepacia*(258/258)  *B. cepacian*(96/97); *B. contaminans*(10/10); *B. diffusa*(12/12)  *B. dolosa*(3/3); *B. gladioli*(9/19); *B. glumae*(7/8); *B. humptydooensis*(3/3)  *B. lata*(4/4); *B. latens*(2/2); *B. mallei*(64/64); *B. metallica*(2/2)  *B. multivorans*(88/88); *B. paludis*(1/1); *B. pseudomallei*(735/739)  *B. pseudomultivorans*(9/9); *B. puraquae*(1/1); *B. pyrrocinia*(5/5)  *B. seminalis*(3/3); *B. stagnalis*(64/64); *B. territorii*(34/34)  *B. thailandensis*(42/42); *B. ubonensis*(88/294); *B. vietnamiensis*(43/43) |
| *aioA/aoxB* | *B. cenocepacia*(6/258); *B. cepacian*(6/97); *B. multivorans*(7/88)  *B. pseudomallei*(7/739); *B. ubonensis*(30/294); *B. vietnamiensis*(12/43) |
| *aioB/aoxA* | *B. cenocepacia*(6/258); *B. cepacian*(3/97); *B. multivorans*(8/88)  *B. pseudomallei*(7/739); *B. ubonensis*(30/294); *B. vietnamiensis*(12/43) |

**Table S6 Distribution of** **Cadmium, zinc, cobalt, copper resistance genes in the genus of *Burkholderia.* The value in parentheses is the number of genomes in which the resistance gene is present and the total number of genomes used for analysis.**

| **Cd, Zn, Co, and Cu resistance gene** | **Species** |
| --- | --- |
| *czc/cusABC* | *B. ambifaria*(9/9); *B. anthina*(8/8); *B. cenocepacia*(258/258)  *B. cepacian*(97/97); *B. contaminans*(10/10); *B. diffusa*(12/12)  *B. dolosa*(3/3); *B. gladioli*(19/19); *B. glumae*(8/8); *B. humptydooensis*(3/3)  *B. lata*(4/4); *B. latens*(2/2); *B. mallei*(64/64); *B. metallica*(2/2)  *B. multivorans*(88/88); *B. plantarii*(1/2); *B. paludis*(2/2)  *B. pseudomallei*(739/739); *B. pseudomultivorans*(9/9); *B. puraquae*(1/1)  *B. pyrrocinia*(5/5); *B. seminalis*(3/3); *B. stabilis*(2/3); *B. stabilis*(3/3)  *B. stagnalis*(64/64)*; B. territorii*(33/34)*; B. thailandensis*(42/42)  *B. ubonensis*(294/294)*; B. vietnamiensis*(43/43) |
| *czcD* | *B. contaminans*(1/10); *B. seminalis*(1/3); *B. stabilis*(1/3); *B. metallica*(1/2) |
| *cop* | *B. ambifaria*(9/9); *B. anthina*(8/8); *B. cenocepacia*(258/258);  *B. cepacian*(97/97); *B. contaminans*(10/10); *B. diffusa*(12/12); *B. dolosa*(3/3)  *B. gladioli*(19/19); *B. glumae*(8/8); *B. humptydooensis*(3/3); *B. lata*(4/4)  *B. latens*(2/2); *B. mallei*(64/64); *B. metallica*(2/2); *B. multivorans*(88/88)  *B. plantarii*(1/2); *B. paludis*(2/2); *B. pseudomallei*(739/739)  *B. pseudomultivorans*(9/9); *B. puraquae*(1/1); *B. pyrrocinia*(5/5)  *B. seminalis*(3/3); *B. stabilis*(2/3); *B. stabilis*(3/3); *B. stagnalis*(64/64)  *B. territorii*(33/34); *B. thailandensis*(42/42); *B. ubonensis*(294/294)  *B. vietnamiensis*(43/43) |

**Table S7. Distribution of Tn7-like components within the genus. * indicates that the strain belongs to Bcc.**

| Species | Tn7-like element | Tn7 like is related to heavy metal resistance |
| --- | --- | --- |
| *B. pseudomallei* | YES | - |
| *B. cenocepacia** | YES | YES |
| *B. ubonensis** | YES | - |
| *B. cepacia** | YES | - |
| *B. multivorans** | YES | YES |
| *B. mallei* | NO | - |
| *B. stagnalis** | YES | - |
| *B. vietnamiensis** | YES | - |
| *B. thailandensis* | YES | - |
| *B. territorii** | YES | - |
| *B. gladioli* | YES | - |
| *B. diffusa** | YES | - |
| *B. contaminans** | YES | YES |
| *B. ambifaria** | NO | - |
| *B. pseudomultivorans** | NO | - |
| *B. anthina** | YES | - |
| *B. glumae* | YES | - |
| *B. pyrrocinia** | NO | - |
| *B. lata** | YES | - |
| *B. humptydooensis* | NO | - |
| *B. seminalis** | YES | - |
| *B. stabilis** | NO | - |
| *B. dolosa** | NO | - |
| *B. latens** | NO | - |
| *B. metallica** | NO | - |
| *B. plantarii* | NO | - |
| *B. singularis* | NO | - |
| *B. catarinensis** | NO | - |
| *B. dabaoshanensis* | YES | - |
| *B. paludis** | NO | - |
| *B. puraquae** | NO | - |

**Table S8. The location of genomic island BGImetal in the genome. * indicates that BGImetal is incomplete and exists on multiple contigs.**

| Species | **BGImetal（chromosme/contig; start-end）** |
| --- | --- |
| *B. contaminans* 293K04B | NQOD01000075.1; 20730-54371 |
| *B. contaminans* LMG 23361 | MCAU02000003.1; 612785-649152 |
| *B. contaminans* 170816* | PQVP01000004.1; PQVP01000005.1 |
| *B. contaminans* AU20979* | PVGK01000099.1; PVGK01000069.1 |
| *B. multivorans* AU15954 | PVFW01000019.1; 3961- 47200 |
| *B. multivorans* AU26250 | PVGY01000031.1; 2435- 45674 |
| *B. multivorans* DDS 15A-1 | CP008728.1; 574969-618208 |
| *B. multivorans* MSMB1272WGS | LPEO01000047.1; 24509- 67748 |
| *B. multivorans* DSOPR54* | NGKK01000224.1; NGKK01000047.1 |
| *B. multivorans* DSOPR57* | NGKL01000053.1; NGKL01000062.1 |
| *B. multivorans* AU22892* | PVGO01000033.1; PVGO01000005.1 |
| *B. cenocepacia* PT15 | NZ_AXZX01000464.1; 20603- 54244 |
| *B. cenocepacia* S2AES | NZ_AXZY01000021.1; 11315- 44956 |
| *B. cenocepacia* VC10178 | NZ_MUQU01000095.1; 23162- 56803 |
| *B. cenocepacia* VC10287 | NZ_MURH01000048.1; 29763- 63404 |
| *B. cenocepacia* VC10878 | NZ_MURI01000024.1; 76857- 110498 |
| *B. cenocepacia* VC11482 | NZ_MUSL01000012.1; 8220- 41864 |
| *B. cenocepacia* VC11575 | NZ_MURJ01000014.1; 214318- 247959 |
| *B. cenocepacia* VC13300 | NZ_MUQV01000198.1; 58933- 92574 |
| *B. cenocepacia* VC13395 | NZ_MUSN01000088.1; 20254- 53896 |
| *B. cenocepacia* VC13450 | NZ_MURL01000197.1; 29823- 63464 |
| *B. cenocepacia* VC14165 | NZ_MUSO01000109.1; 20553- 54195 |
| *B. cenocepacia* VC14507 | NZ_MUQW01000039.1; 116897- 150538 |
| *B. cenocepacia* VC14543 | NZ_MURN01000158.1; 126810- 160451 |
| *B. cenocepacia* VC14636 | NZ_MUSP01000098.1; 8202- 41844 |
| *B. cenocepacia* VC15221 | NZ_MUSR01000175.1; 13267- 46909 |
| *B. cenocepacia* VC15235 | NZ_MURO01000051.1; 29727- 63368 |
| *B. cenocepacia* VC3868 | NZ_MURA01000153.1; 70571- 104212 |
| *B. cenocepacia* VC3929 | NZ_MUQL01000031.1; 116839- 150480 |
| *B. cenocepacia* VC4480 | NZ_MURB01000220.1; 56091- 89732 |
| *B. cenocepacia* VC4558 | NZ_MUJO01000009.1; 1451252- 1484893 |
| *B. cenocepacia* VC5300 | NZ_MURC01000058.1; 154489- 188130 |
| *B. cenocepacia* VC5731 | NZ_MUQN01000004.1; 110428- 144069 |
| *B. cenocepacia* VC5732 | NZ_MUQM01000160.1; 116739- 150380 |
| *B. cenocepacia* VC6903 | NZ_MUQP01000108.1; 116777- 150418 |
| *B. cenocepacia* VC6904 | NZ_MUQO01000169.1; 23273- 56914 |
| *B. cenocepacia* VC6916 | NZ_MUSE01000114.1; 20333- 53977 |
| *B. cenocepacia* VC6917 | NZ_MUSF01000049.1; 8189­- 41832 |
| *B. cenocepacia* VC7378 | NZ_MUSG01000009.1; 20553- 54196 |
| *B. cenocepacia* VC7603 | NZ_MUQQ01000029.1; 116815- 150456 |
| *B. cenocepacia* VC7604 | NZ_MUJP01000005.1; 1447227-1480868 |
| *B. cenocepacia* VC7884 | NZ_MUSI01000099.1; 13381- 47028 |
| *B. cenocepacia* VC8057 | NZ_MUQR01000181.1; 23190- 56831 |
| *B. cenocepacia* VC8556 | NZ_MUQS01000052.1; 116609- 150250 |
| *B. cenocepacia* VC8946 | NZ_MURE01000079.1; 69664- 103305 |
| *B. cenocepacia* VC8947 | NZ_MURF01000120.1; 73175- 106816 |
| *B. cenocepacia* VC9612 | NZ_MUQT01000045.1; 21309- 54950 |
| *B. cenocepacia* VC9670 | NZ_MURG01000002.1; 29828- 63469 |
| *B. cenocepacia* VC9970 | NZ_MUSK01000104.1; 13302- 46947 |
| *B. cenocepacia* VC7379 | NZ_MUSH01000129.1; 20430- 54073 |
| *B. cenocepacia* VC10394* | NZ_MUTE01000093.1; NZ_MUTE01000024.1 |
| *B. cenocepacia* VC11261* | NZ_MUTF01000081.1; NZ_MUTF01000001.1 |
| *B. cenocepacia* VC11903* | NZ_MUTG01000051.1; NZ_MUTG01000078.1 |
| *B. cenocepacia* VC12229* | NZ_MUSM01000016.1; NZ_MUSM01000148.1; NZ_MUSM01000040.1 |
| *B. cenocepacia* VC13112* | NZ_MUTH01000042.1; NZ_MUTH01000020.1 |
| *B. cenocepacia* VC14637* | NZ_MUSQ01000043.1; NZ_MUSQ01000194.1 |
| *B. cenocepacia* VC14761* | NZ_MUTI01000070.1; NZ_MUTI01000001.1 |
| *B. cenocepacia* VC14762* | NZ_MUTJ01000015.1; NZ_MUTJ01000057.1 |
| *B. cenocepacia* VC15305* | NZ_MUTK01000019.1; NZ_MUTK01000083.1 |
| *B. cenocepacia* VC7292* | NZ_MUTB01000015.1; NZ_MUTB01000038.1 |
| *B. cenocepacia* VC7530* | NZ_MUTC01000138.1; NZ_MUTC01000134.1 |
| *B. cenocepacia* VC8945* | NZ_MURD01000024.1; NZ_MURD01000028.1 |
| *B. cenocepacia* VC9855* | NZ_MUTD01000036.1; NZ_MUTD01000021.1 |
| *B. cenocepacia* 79 BCEN* | NZ_JUSV01002379.1; NZ_JUSV01002454.1 |
| *B. cenocepacia* BC-19* | NZ_JYMX01000131.1; NZ_JYMX01000077.1; NZ_JYMX01000040.1 |
| *B. cenocepacia* BC-21* | NZ_JYMY01000097.1; NZ_JYMY01000023.1; NZ_JYMY01000040.1 |
| *B. cenocepacia* BC-40* | NZ_JYMZ01000125.1; NZ_JYMZ01000126.1; NZ_JYMZ01000001.1 |
| *B. cenocepacia* BC-41* | NZ_JYNA01000095.1; NZ_JYNA01000074.1; NZ_JYNA01000011.1 |
| *B. cenocepacia* VC12599* | NZ_MURK01000044.1; NZ_MURK01000086.1 |
| *B. cenocepacia* VC14013* | NZ_MURM01000147.1; NZ_MURM01000157.1 |
| *B. cenocepacia* VC14748* | NZ_MUQX01000143.1; NZ_MUQX01000034.1 |
| *B. cenocepacia* VC14815* | NZ_MUQY01000002.1; NZ_MUQY01000205.1 |
| *B. cenocepacia* VC15049* | NZ_MUQZ01000003.1; NZ_MUQZ01000042.1 |
| *B. cenocepacia* VC15566* | NZ_MURP01000084.1; NZ_MURP01000091.1 |
| *B. cenocepacia* VC8222* | NZ_MUSJ01000053.1; NZ_MUSJ01000009.1; NZ_MUSJ01000073.1 |


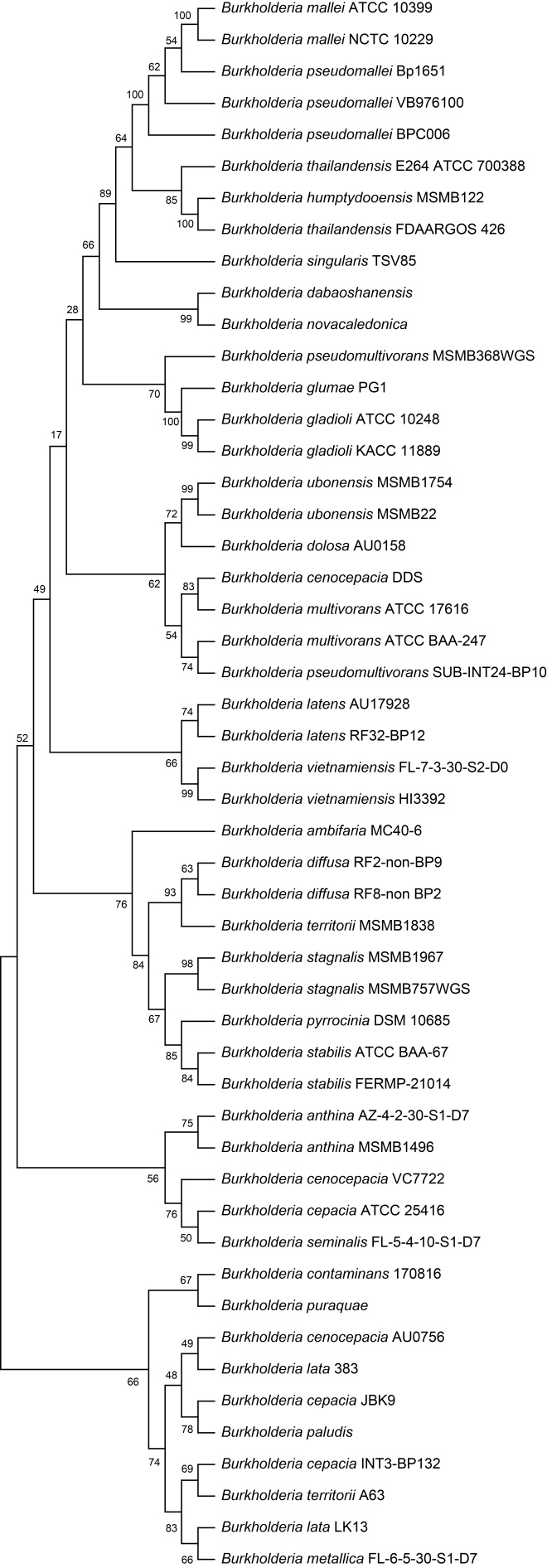


**Figure S2. The 16s rDNA phylogenetic tree of 60** ***Burkholderia* genomes.**


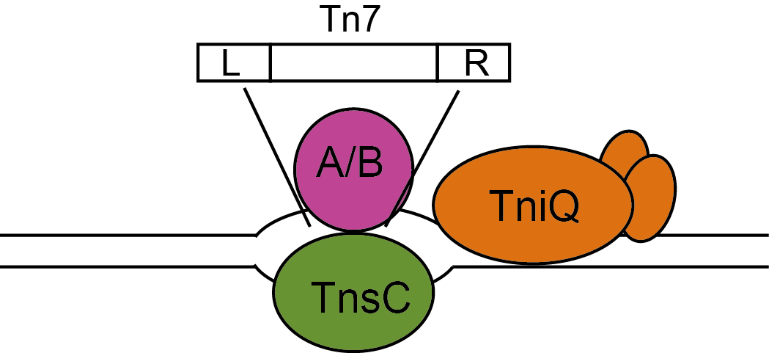


**Figure S3 Model of Tn7 transposition. Targeting a specific binding site (attTn7) of TniQ leads to asymmetric aberration of DNA, thereby recruiting TnsC to bind to the target site. TnsA/TnsB transposase complexes with the target protein to activate transcription and translocate Tn7 into the genome.**
